# A Class of Diacylglycerol Acyltransferase 1 Inhibitors Identified by a Combination of Phenotypic High-throughput Screening, Genomics, and Genetics

**DOI:** 10.1016/j.ebiom.2016.04.014

**Published:** 2016-04-16

**Authors:** Kirsten Tschapalda, Ya-Qin Zhang, Li Liu, Kseniya Golovnina, Thomas Schlemper, Thomas O. Eichmann, Madhu Lal-Nag, Urmila Sreenivasan, John McLenithan, Slava Ziegler, Carole Sztalryd, Achim Lass, Douglas Auld, Brian Oliver, Herbert Waldmann, Zhuyin Li, Min Shen, Matthew B. Boxer, Mathias Beller

**Affiliations:** a Systems Biology of Lipid Metabolism, Heinrich Heine University Düsseldorf, Germany; b Institute for Mathematical Modeling of Biological Systems, Heinrich Heine University Düsseldorf, Germany; c Department of Chemical Biology, Max Planck Institute of Molecular Physiology, Dortmund, Germany; d Department of Molecular Developmental Biology, Max Planck Institute for Biophysical Chemistry, Göttingen, Germany; e National Center for Advancing Translational Sciences, National Institutes of Health, Rockville, USA; f National Institute of Diabetes, Digestive and Kidney Diseases, National Institutes of Health, Bethesda, USA; g Institute of Molecular Biosciences, University of Graz, Austria; h Department of Medicine, Division of Endocrinology University of Maryland School of Medicine, USA; i Baltimore VA Medical Center, VA Research Service, Geriatric Research, Education and Clinical Center (GRECC) and VA Maryland Health Care System, Department of Medicine, Division of Endocrinology University of Maryland School of Medicine, USA

## Abstract

Excess lipid storage is an epidemic problem in human populations. Thus, the identification of small molecules to treat or prevent lipid storage-related metabolic complications is of great interest. Here we screened > 320.000 compounds for their ability to prevent a cellular lipid accumulation phenotype. We used fly cells because the multifarious tools available for this organism should facilitate unraveling the mechanism-of-action of active small molecules. Of the several hundred lipid storage inhibitors identified in the primary screen we concentrated on three structurally diverse and potent compound classes active in cells of multiple species (including human) and negligible cytotoxicity. Together with Drosophila in vivo epistasis experiments, RNA-Seq expression profiles suggested that the target of one of the small molecules was diacylglycerol acyltransferase 1 (DGAT1), a key enzyme in the production of triacylglycerols and prominent human drug target. We confirmed this prediction by biochemical and enzymatic activity tests.

## Highlights

• We identified > 600 potent small molecule inhibitors of cellular lipid storage deposition.
• RNA-Seq expression profiling discriminated the activity of three lead scaffolds and guided subsequent functional studies.
• We discovered a class of DGAT1 inhibitors, which is active in fly and mammalian cell lines as well as whole flies.

Obesity and other lipid storage associated diseases are a growing health threat of human populations. In an undirected phenotypic screen, we identified pharmacologically active small molecules that reduce or enhance lipid storage. Our work focuses on three lead structures that prevent lipid storage in diverse cellular systems including cells from a diabetes patient. In order to elucidate the compound mechanisms-of-action and cellular targets, we used a combination of RNA-Seq transcriptional profiling and diverse functional assays. Our results strongly suggest that one of our lead structures represents a class of inhibitors targeting the key lipogenic enzyme diacylglycerol acyltransferase 1.

## 1 Introduction

Cells of organisms from all phyla balance fluctuations in energy demand and supply by the regulated deposition and remobilization of energy rich storage molecules. Among these, the neutral lipids are the most efficient. Neutral lipid synthesis relies on the conversion of surplus fatty acids by multiple biochemical pathways into triacylglycerols (TAG) and sterol esters (SE). TAG and SE are stored in specialized cellular organelles, called lipid droplets (LDs) (Farese and Walther, 2009, Murphy, 2001). LDs consist of a hydrophobic core harboring the storage lipids surrounded by a phospholipid monolayer and associated proteins. In addition to the energy buffering function, the conversion of fatty acids to mono-, di-, and finally TAG protects cells from lipotoxicity. Additionally, lipids stored within LDs also serve as anabolic building blocks for membrane, hormone, and other synthesis processes. While the molecular details of the regulated lipid storage remobilization in adipocytes are emerging (Lass et al., 2011), many details of cellular lipid metabolism remain elusive.

In healthy individuals, lipid storage amounts rely on the balance between energy intake and expenditure. However, modern lifestyles are marked by the excessive intake of high caloric food and only limited physical activity. Additionally, genotype, epigenetic effects and the gut microbiome affect energy storage (Carone et al., 2010, Farooqi and O’Rahilly, 2007, Lusis et al., 2008, Sullivan and Grove, 2009, Turnbaugh et al., 2006). These intrinsic and extrinsic factors have resulted in a dramatic increase in the number of overweight and obese individuals, which has created a global epidemic. Aberrant lipid storage amounts (high or low) are associated with or cause many other diseases. For example, obesity co-morbidities include increased susceptibility to diabetes, atherosclerosis, cancer, or infections (Gross and Silver, 2014). In addition to obesity, ectopic lipid deposition of the liver (liver steatosis), muscle or glia cells of individuals facing neurodegeneration (Liu et al., 2015, Shulman, 2014, Zámbó et al., 2013) as well as the replication of certain pathogens including chlamydia or Hepatitis C virus (Boulant et al., 2008, Kumar et al., 2006) depend on LDs. Thus, there is great need for pharmacological approaches to understand and treat a host of lipid storage phenotypes.

Phenotypic screening approaches are commonly used to identify small molecules affecting processes where the molecular details are unknown (Eder et al., 2014). In some cases, it is desirable to understand the molecular mechanism-of-action of compounds derived from phenotypic screens but mapping the activity of compounds to molecular targets presents a significant screening bottleneck. Different strategies have been used for this difficult task including complementary RNAi screening for phenocopies (Gonsalves et al., 2011, Perrimon et al., 2007, Winter et al., 2011) or biochemical target identification strategies using affinity enrichment experiments coupled to the mass spectrometry based identification of bound proteins (Vendrell-Navarro et al., 2015, Ziegler et al., 2013).

Here we used the non-mammalian model system Drosophila to identify small molecule inhibitors of cellular lipid deposition by a phenotypic high-throughput screen. The Drosophila genome harbors many of the same genes regulating metabolism as humans (Baker and Thummel, 2007) and has developed into a vital tool in metabolic research (Kühnlein, 2011, Pandey and Nichols, 2011, Rajan and Perrimon, 2011). While there are obvious differences in the life histories of mammals and flies, as well as fly-specific metabolic details such as sterol auxotrophy (Carvalho et al., 2010) and the absence of an omega-3 and omega-5 fatty acid requirement (Shen et al., 2010) there are many more similarities that make Drosophila an attractive model. For example, genes encoding lipid metabolic enzymes and regulators (Buszczak et al., 2002, Tian et al., 2011, Wilfling et al., 2013) as well as important hormones such as insulin (Böhni et al., 1999), glucagon (Grönke et al., 2007), and leptin (Rajan and Perrimon, 2012) are functionally conserved in the fly. It is increasingly clear that LD cell biology is essentially the same in flies and mammals, as for example homologous PERILIPIN proteins coat (Beller et al., 2010, Grönke et al., 2003), and lipases dock (Bi et al., 2012, Grönke et al., 2005), with LDs in both organisms.

To take full advantage of the unparalleled genetic tools available in Drosophila to develop small molecules that inhibit lipid storage, we used a tiered experimental approach that relied on a primary screen in Drosophila cells, followed by the generation of derivatives of selected top candidate structural scaffolds for testing in a range of mice, monkey, and human cells. Those small molecules showing reduced lipid storage in these cells were further analyzed by functional assays and RNA-Seq based transcriptional profiling to determine what pathways were altered. By this effort, we could map the activity of one group of small molecules to the inhibition of the lipogenic diacylglycerol acyltransferase 1 (DGAT1) enzyme, which is a known human obesity drug target.

## 2 Materials & Methods

All methods are described in detail in the Supplemental Experimental Procedures section.

### 2.1 qHTS Screening and Chemical Syntheses of Small Molecule Derivatives, Oleic Acid Feeding and Small Molecule Treatment of Cells

Drosophila and mammalian cells were kept in the appropriate medium supplemented with 5% FCS and the indicated amount of small molecule or DMSO solvent alone. For the quantitative high-throughput small molecule screen (qHTS; PubChem Assay ID 2685), S3 cells were dispensed in 1536-well plates and treated with multiple concentrations of each library compound (46 μM to 3 nM in 1:5 dilution steps). LDs were induced by the addition of 400 μM OA bound to fatty acid free BSA. After 18 h of treatment, the cells were fixed and stained for LDs with the hydrophobic dye BODIPY 493/503 and the Cell Tracker Red CMTPX dye (both from Molecular Probes/Invitrogen) to enumerate cell number. Fluorescence was detected by excitation of the fluorophores with a 488 nm laser on an Acumen Explorer (TTP Lab Tech, Hertfordshire, UK) and quantification of the lipid storage levels was normalized to the cell number.

To test for defects in cholesteryl-ester LD deposition in Drosophila S3 or murine AML12 cells, we added either NBD-cholesterol (Molecular Probes/Invitrogen) alone or in combination with OA in the presence of the indicated amounts of small molecule or the solvent DMSO alone. Experiments were analyzed by fluorescence microscopy.

In addition to BODIPY493/503 we also used the lipophilic dye HCS LipidTOX deep red to stain LDs and counted cells based on DNA/nuclei signals achieved by Hoechst or DAPI staining. Images were either qualitatively scored by visual inspection or lipid storage amounts were quantified using custom CellProfiler (Kamentsky et al., 2011) image segmentation algorithms. As an alternative to image-based readouts, lipids were extracted from the cells and analyzed by thin layer chromatography or HPLC-ELSD.

### 2.2 Measurement of Fatty Acid Uptake

Drosophila S3 cells were incubated with the fluorescent C12 fatty acid tracer QBT fluorescent fatty acid uptake reagent (Molecular Devices) according to the manufacturer's description. Cells were subsequently analyzed by confocal microscopy.

For the COS7 cell experiments, the cells received a short pulse of a mixture of cold and ^3^H-labeled OA in the presence of the indicated small molecule amount or the DMSO solvent alone. The cells were rapidly washed and the internalized radioactivity was quantified by scintillation counting.

### 2.3 Next Generation Sequencing Experiments Including Data Analysis

We treated 36 samples of Drosophila S3 cells with 1 μM of the respective inactive or active compounds for four hours in serum-reduced medium including 5% FCS. Multiple controls for the effect of OA and DMSO were included. Each sample was measured at least in triplicate. Following treatment, the total RNA was extracted from the cells and processed for RNA-Seq experiments. Details concerning the treatment of the cells, library preparation, sequencing, data analysis and quality control measures are described in depth in the Supplemental Experimental Procedures and in GEO accession GSE67503.

### 2.4 Fatty Acid Beta-oxidation Measurements

Fatty acid β-oxidation was measured as previously described (Wang et al., 2009, Wang et al., 2011) using metabolic radioactive labeling of murine AML12 cells grown in the presence of DMSO solvent only or the indicated small molecule. Both CO_2_ production and the amount of acid soluble metabolites (ASM) were quantified.

### 2.5 Drosophila Experiments

Drosophila were kept on a standard cornmeal/molasses diet for propagation. For compound treatment experiments we included 10 to 20 μM of the small molecule or the DMSO solvent only (final concentration 0.2% DMSO) in food which consisted of: 0.624 g Agar, 8 g polenta, 1 g soy flour, 1.8 g dry yeast, 2.2 g treacle, 8 g malt extract and 1.5 mL nipagin solution (10% in Ethanol) and 0.63 mL propionic acid per 100 mL. Food was cooled to approximately 60 °C before we added the compound and/or DMSO and was aliquoted to the fly vials where it solidified. Parental flies were placed on this food and allowed to lay eggs for three days before they were removed. The compound-treated flies we analyzed thus completed their whole development in the presence of compound and/or DMSO. We collected larvae, freshly eclosed flies, and six day post eclosion adults and measured their triglyceride content using established protocols (Hildebrandt et al., 2011). High-fat diet treatment was done by the addition of 10% coconut oil (Sigma-Aldrich) to the food and followed established protocols (Shirazi et al., 2014). Details of the fly experiments as well as information concerning the different fly strains used are provided in the Supplemental Experimental Procedures section.

## 3 Results

### 3.1 A Phenotypic, Quantitative High-throughput Screen Identifies Potent Inhibitors of Lipid Deposition

To identify small molecules reducing cellular lipid storage amounts we used a quantitative-high throughput image-based assay to measure LD number per cell in the presence of excess free fatty acid in Drosophila cells. In the absence of exogenous free fatty acids, the embryonic Drosophila Kc167 and S3 cell lines store little lipid. However, when we added free fatty acids in the form of oleic acid (OA) to the media, both cell lines accumulated LDs within 16 h (Figure S1a–c). As S3 cells showed higher lipid storage capacity, and thus an elevated dynamic range and more robust response, we used these cells for the screening campaign.

**Fig. 1:**
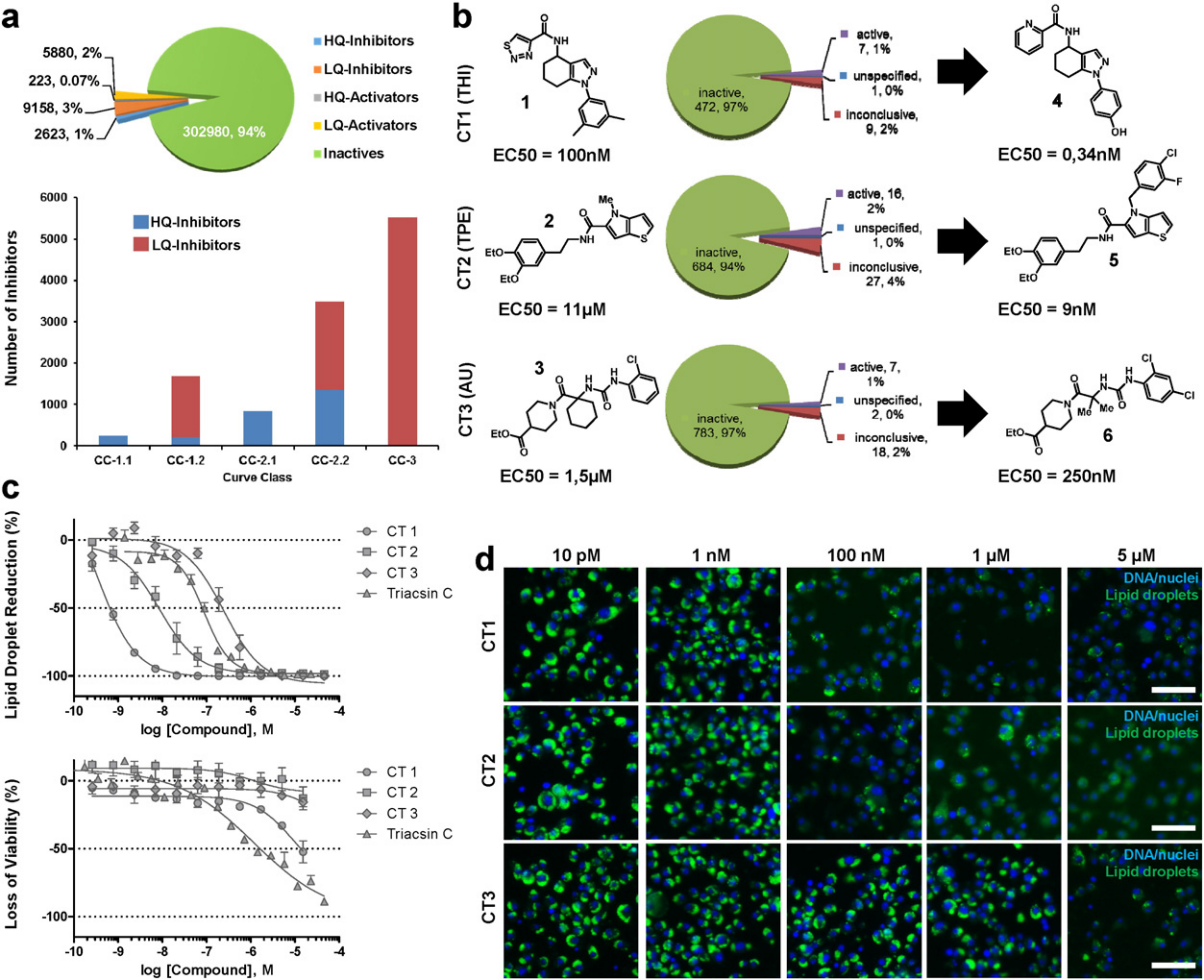
qHTS screen identifies potent lipid storage inhibitors. (a) Summary of the qHTS screening results. The MLSMR compound library was tested for lipid storage inhibitors on Drosophila S3 cells incubated with 400 μM OA. All structures were tested in six different concentrations and dose-response activity relationships were calculated for each compound. Based on the presence or absence of the characteristics of a classical sigmoidal dose-response curve, activities were classified (compare to qHTS introduction and curve class (CC) classification described in: (Inglese et al., 2006)). Only high-quality inhibitors showing a CC 1 (perfect sigmoidal shape) or CC 2 (sigmoidal shape lacking one of the asymptotes) were considered in the following. The bar plot provides information concerning the number of high- and low quality inhibitors broken down to compound curve classes. 248 compounds were classified as curve class 1.1 (cc-1.1), 1682 compounds belonged to cc-1.2 (203 high-quality and 1479 low-quality hits), cc-2.1 contained 831 high-quality inhibitors, 3488 compounds were classified as cc-2.2 (1341 high-quality and 2147 low-quality hits), and 5532 low-quality inhibitors belonged to cc-3. (b) We concentrate on three structurally diverse chemical scaffolds (“chemotypes”; CT) represented by structures 1 to 3, which resulted in low lipid storage. The structures are characterized by a tetrahydroindazole (CT1; THI), thienopyrrole (CT2; TPE) or arylureido (CT3; AU) core. The pie charts depict that the structures showed no activity in most other MLSMR screens, suggesting specificity for the lipid storage phenotype. The synthesis of analogs in the course of structure–activity relationship (SAR) studies resulted in highly active structures (structures 4 to 6). Derivative nomenclature is based on the structure core abbreviation followed by a unique number separated by a dash (compare with Figure S2). (c) The optimized structures resulted in a dosage-dependent reduction of cellular lipid storage levels and affected viability only at extremely high concentrations (CT1 = THI-4; CT2 = TPE-5 and CT3 = AU-6; data is given as mean ± standard error of the mean (SEM); *n* = 2 wells). (d) Drosophila S3 cells incubated with 600 μM OA and the indicated concentrations of CT1 (THI-4), CT2 (TPE-5) and CT3 (AU-6) overnight. Also see Figure S1. Scale bars in panel (d) represent 50 μm.

Following exploratory screens with the Library of Pharmacologically Active Compounds (LOPAC; Sigma-Aldrich), Tocris (Tocris Bioscience), and the National Institutes of Health (NIH) Chemical Genomics Center (NCGC) Pharmaceutical Collection (Huang et al., 2011) (data not shown), we screened 320,864 small molecules from the Molecular Libraries Small Molecule Repository in six concentrations spanning four orders of magnitude (Pubchem Bioassay ID 2685). In total, we identified 2623 high-quality lipid storage inhibitors that showed a strong quantitative effect on LDs (Fig. 1a). For the classification of active small molecules, we used a combination of filtering criteria including efficacy, concentration dependency (curve class), low cytotoxicity, and the presence of multiple structurally similar compounds. This procedure followed the “Quantitative high-throughput screening (qHTS) strategy” (Inglese et al., 2006). In addition to the 2623 high-quality lipid storage inhibitors, we also identified 223 high-quality lipid storage activators. Finally, we identified 9158 inhibitors and 5880 activators with low quality (Fig. 1a).

We concentrated on three unique chemical scaffolds, or chemotypes (CT), based on tetrahydroindazole (CT1; THI), thienopyrrole (CT2; TPE) or arylureido (CT3; AU) cores. These small molecules were not frequent hits in previous Molecular Libraries Program screens (Fig. 1b), suggesting a potential for specificity. In order to guide the development of more potent CT derivatives, we undertook a medicinal chemistry effort to better understand structure activity relationships (SAR). In total, we synthesized 89 structural variants (Figure S2) and obtained potent and non-toxic modulators of lipid stores with EC50 values in the pM–nM range (Fig. 1b–d and Figure S1d–f). In comparison to the known acyl-CoA synthase inhibitor TriacsinC (Igal et al., 1997), which we used as a positive control, two of the three CTs showed a superior efficacy with half maximal effective concentrations (EC50) of 90 nM for TriacsinC and 0.34 and 9 nM for CT1 and CT2, respectively (Fig. 1b,c).

### 3.2 The activity of the Identified Lipid Storage Inhibitors is Evolutionary Conserved

**Fig. 2:**
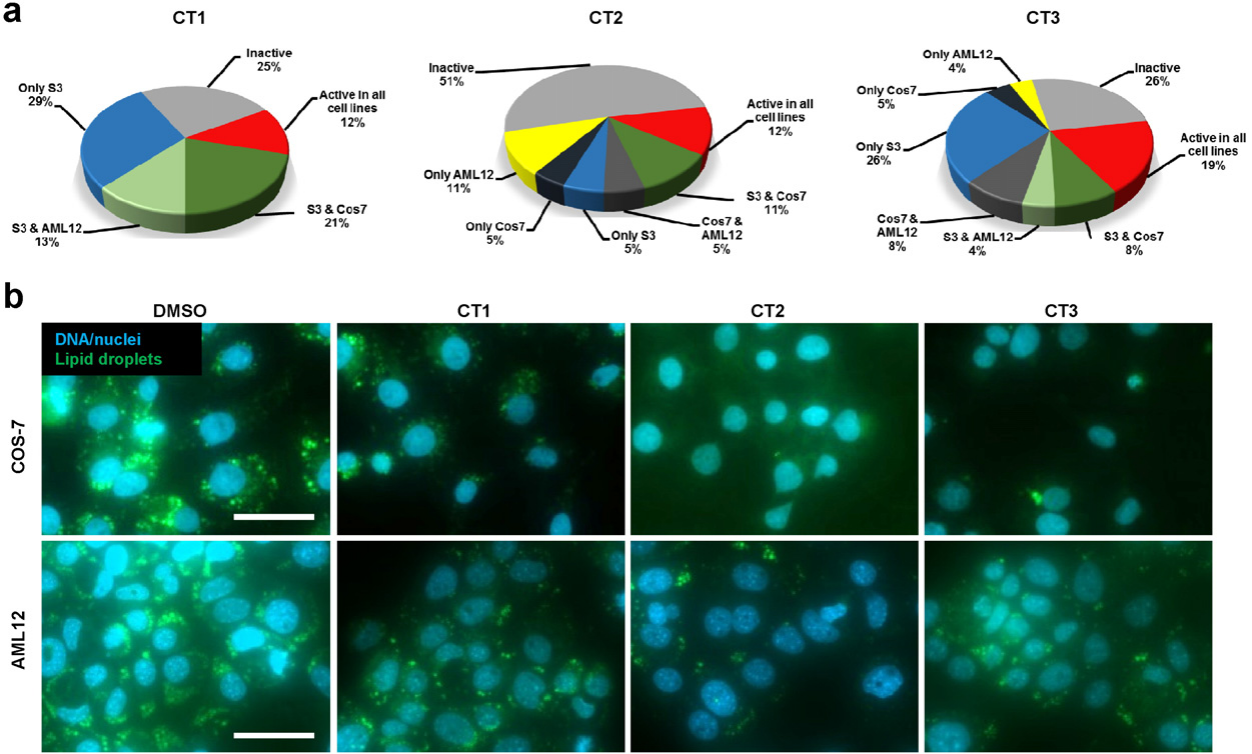
The activity of the three chemotypes is evolutionary conserved. (a) The SAR structures were tested in COS7 monkey kidney cells and murine AML12 hepatocytes for an evolutionary conserved function. 12–19% of the structural variants showed activity across all cell lines. (b) Example of the lipid storage phenotype in COS7 and AML12 cells caused by treatment with 5 μM CT1 (THI-4), CT2 (TPE-5) and CT3 (AU-6) in the presence of 400 μM and 200 μM OA, respectively. Also see Figure S2. Scale bars in panel (b) represent 50 μm.

To be an effective tool or lead compound for drug development, it was important that these analogs were potent modulators of lipid storage in mammalian cells. We chose monkey COS7 kidney and mouse AML12 hepatocytes to test all 89 generated derivatives (Fig. 2 and Figure S2). In the presence of OA, 12–19% of the derivatives of each CT showed activity in both mammalian and fly cell lines (Fig. 2 and Figure S2). Additionally, we identified small molecules with species-specific activities as well as analogs with low activities across all cell types. These data suggest that a SAR approach maximizes conserved phenotypic responses to CTs.

### 3.3 Expression Profiling Guides Chemotype Classification

**Fig. 3:**
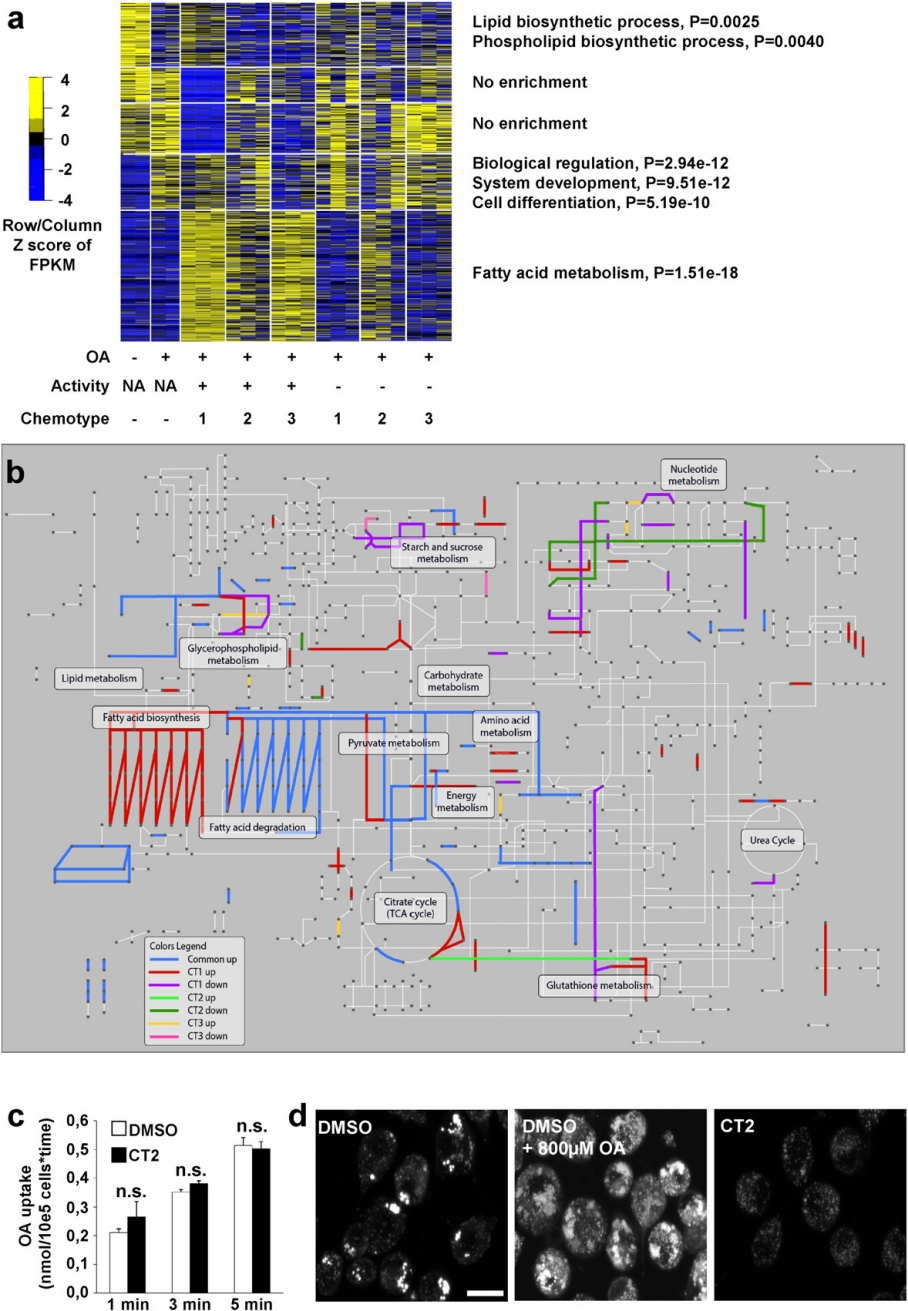
The different CTs provoke diagnostic transcriptional responses and the uptake of fatty acids is unaffected by CT2. We investigated the transcriptional response of S3 cells after 4 h of CT treatment in the presence or absence of 200 μM OA with either 1 μM active (CT1 = THI-4, CT2 = TPE-69, or CT3 = AU-6) or 1 μM inactive (CT1 = THI-68, CT2 = TPE-67, or CT3 = AU-73) compound derivatives. (a) Expression profiles showing differentially expressed (DE) genes between treated and control samples. The scaled DE gene expression values (FPKM), Z scores, are presented in 5 k-means clusters of genes (rows). (b) *Drosophila melanogaster* specific KEGG metabolic pathways with highlighted reactions involved in small molecule responses (see color legend). (c) COS7 cells were kept in medium containing either DMSO, or 5 μM TPE-5 in DMSO. The cells were subsequently loaded with radiolabeled fatty acids, the cells were washed and internalized radioactivity was quantified by scintillation counting (bars provide mean ± standard deviation (s.d.), *n* = 3 wells). (d) Confocal images of Drosophila S3 cells incubated with the QBT-fluorescent fatty acid uptake reagent (Molecular Devices). On top of the fatty acid uptake reagent, the cell medium was either additionally supplemented with DMSO, DMSO and 800 μM OA or 5 μM TPE-5 solved in DMSO. Scale bar represents 10 μm. The following significance levels are used: not significant (n.s.) *p* ≥ 0.05; *p < 0.05; **p < 0.01; ***p < 0.001. Results between two independent groups were determined by Student's *t*-test. Also see Figures S3, S4 and Tables S1–S5 in the online version at http://dx.doi.org/10.1016/j.ebiom.2016.04.014.

Cells show prominent transcriptional changes as a result of metabolic changes in nutrient availability or stress (Choi et al., 2012, De Gottardi et al., 2007, De Nadal et al., 2011). We hypothesized that changes in gene expression signatures could focus our attention on specific pathways as well as guide classification of CT effects on metabolism. CTs with a similar mechanism-of-action should share gene expression signature aspects. We therefore performed RNA-Seq experiments on S3 cells after 4 h of treatment (Fig. 3a,b and Figures S3 & S4; for details see Supplemental Methods Section, Supplemental Table S1, Table S2, Table S3) to determine if the three CTs had a similar effect on gene expression, or whether there were expression signatures consistent with distinct mechanisms of action.

In addition to active CT derivatives we also included derivatives showing no or only little activity. This way we could identify CT-specific gene expression changes unrelated to the lipid storage phenotype and thus representing a more general response to the CT structure as well as potential off-target effects. When we quantified the number of differentially expressed genes between the DMSO treated control and active or inactive CT derivatives (Figure S4a; *p*-value < 0.001) respectively, treatment with the active small molecule derivatives resulted in many more differentially expressed genes as compared to the controls. Thus, gene expression profiling appears to be diagnostic for the compound-induced lipid storage phenotype.

When we clustered the expression measures of genes that were differentially expressed between at least two treatments, we obtained five clusters (Fig. 3a, Figure S4; Table S4). In OA-fed control cells, expression of cluster 1 genes was lower than in cells without exogenous free fatty acids. Cluster 1 was enriched in genes encoding enzymes that synthesize lipids and phospholipids based on Gene Ontology (GO) analysis (Fig. 3b, Figure S4d, Table S4, Table S5), consistent with down-regulation of fatty acid synthesis in the presence of an exogenous supply. Clusters 2 and 3 were similarly expressed in non-compound-treated control cells with or without OA and showed no obvious link to lipid metabolism based on GO term analysis. These genes showed reduced expression in cells treated with CT1 or CT3, raising the possibility of a more general cellular response or off-target effects. Cluster 4 genes were upregulated by OA addition and enriched for genes encoding regulatory functions during differentiation (Figure S4d). These genes showed modestly decreased expression following treatment with CT3. Cluster 5 was highly enriched in genes that encode proteins involved in shunting lipids to other pathways, such as the TCA cycle, alpha- and beta-oxidation (Figure S4d) and was upregulated following treatment with all three CTs. This suggests that the cellular phenotype of reduced lipid storage was achieved not by blocking fatty acid transport into cells, but by enhanced catabolism. To test the immediate effect of compound activity on the fatty acid uptake (e.g. by competitive effects during the fatty acid transport), we performed radiolabeled fatty acid uptake experiments (Fig. 3c). Here, we noted no significant difference as compared to control cells. When we provided fluorescently labeled fatty acids to DMSO treated control cells (Fig. 3d) they were successfully incorporated in LDs. Cells which received on top of the fluorescent fatty acids unlabeled oleic acid showed more prominent lipid stores (middle picture in Fig. 3d). CT2 treated cells also incorporated the fluorescently labeled fatty acids in LDs (Fig. 3d). However, the total fluorescence intensity was lower as compared to the DMSO treated control cells, probably because of the reduced lipid storage phenotype. These data indicate that CT treatment indeed has little to no impact on the internalization of fatty acids. Thus, to achieve the low lipid storage phenotype, fatty acids must be shunted into other pathways to convert excess free fatty acids to energy production, possibly to protect from lipotoxicity.

While we observed a core set of changes consistent with rerouting lipids to energy production, there were also CT specific changes in expression (Fig. 3, Figure S4e). CT1 treated cells showed increased expression of genes encoding both anabolic and catabolic lipid metabolism functions (Fig. 3a,b). These changes in gene expression are consistent with a simultaneous increase in lipid synthesis (e.g. the fatty acid synthase *CG3523*; (Pierre et al., 2014)), and increased lipolysis (e.g. *brummer* lipase; (Grönke et al., 2005)) from LDs (compare Table S1). This suggests a futile cycle of lipid synthesis and breakdown. CT2 treated cells showed more restricted changes in gene expression among genes that also respond to OA loading (clusters 2–4; Fig. 3a), suggesting a role in blocking lipid production. CT3 resulted in upregulated expression of genes involved in the xenobiotic response such as Glutathione-S-transferases (mgstl, Gst T1, Gst T2, Gst T3 and GstE2) or ATP-coupled export proteins (CG2316) that may result in biological clearance (Table S1), in addition to reduced expression of genes in clusters 2–4.

The limited number of CT2 specific gene expression changes (23 genes were differentially regulated; other changes can be explained by the addition of OA; please compare Table S3) were in agreement with a limited and specific effect of CT2 on lipid metabolism. Therefore, we concentrated our efforts on this CT.

### 3.4 Phenotypic Analysis of CT2 Treated Cells

**Fig. 4:**
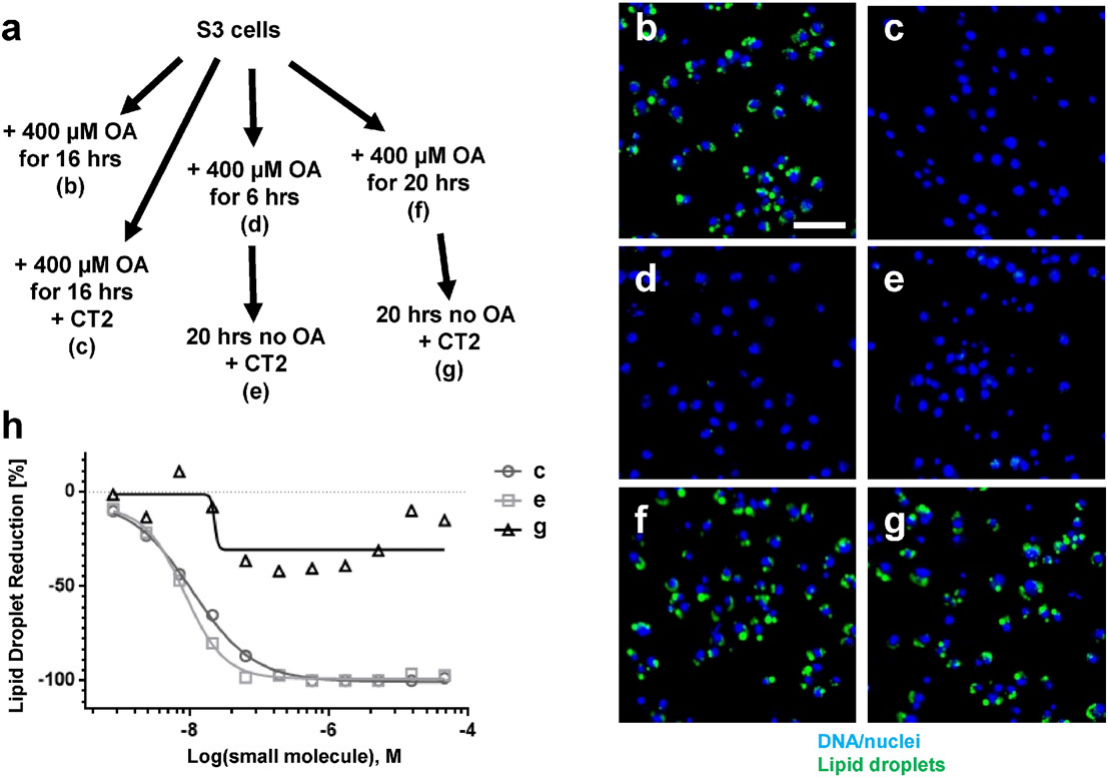
CT2 does not act via increased lipolysis. (a) Experimental outline of CT2 feeding (all conditions: 5 μM TPE-5) regimens in Drosophila S3 cells and cross-references to the corresponding images shown in (b–g). (h) Dose-response curves to the conditions shown in (c, e, g). Points represent single well measurements. Lipid droplets are stained with BODIPY493/503 and Nuclei/DNA with DAPI. Scale bar represents 50 μm.

Because we determined that free fatty acids were normally imported in CT2 treated cells (Fig. 3c,d), we next asked if CT2 slowed down TAG synthesis or accelerated TAG breakdown. To study breakdown we treated pre-OA loaded cells with CT2 (Fig. 4a, d–g). To study synthesis, we treated the cells with OA and CT2 at the same time (Fig. 4a–c). We observed that CT2 blocked accumulation of lipid in droplets (Fig. 4; dose-response information for conditions c, e, and g shown in panel h), but did not reduce lipid storage once prominent LDs were formed, consistent with a block in TAG synthesis.

**Fig. 5:**
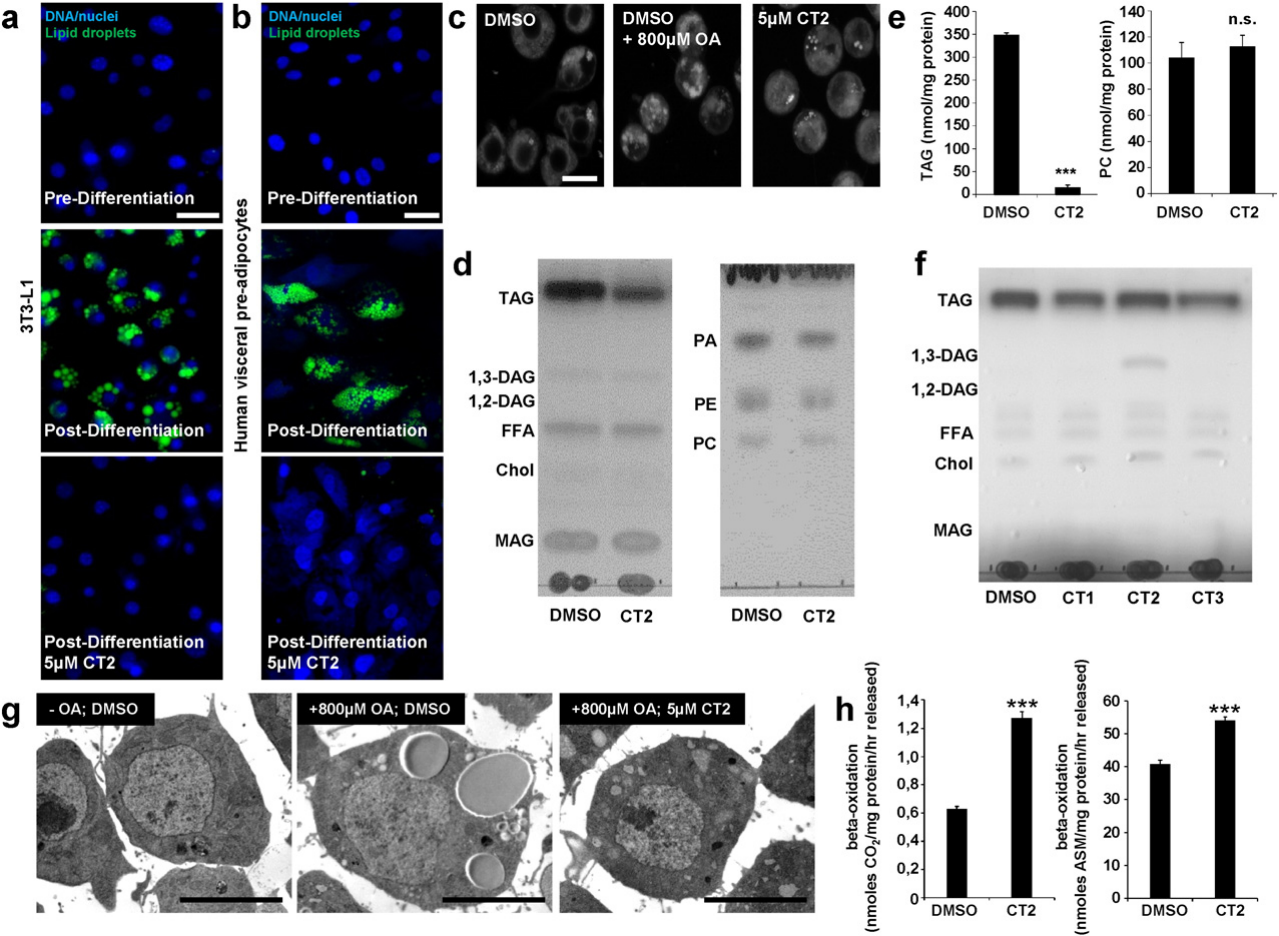
Detailed analysis of the CT2 induced lipid storage phenotype. (a) Murine 3T3-L1 or (b) human visceral pre-adipocytes were differentiated into adipocytes in the presence of 5 μM CT2 (TPE-5) which results in blocked lipid deposition. DNA is shown in blue (DAPI) and neutral lipids in green (BODIPY 493/503). (c) Drosophila Kc167 cells incubated with 1 μg/mL NBD-cholesterol in the presence of DMSO only, DMSO and 800 μM OA or 5 μM CT2 (TPE-5) solved in DMSO. CT2 does not affect the deposition of cholesterylester droplets. (d) Thin layer chromatography analysis of lipid extracts of Kc167 cells treated with 5 μM of CT2 (TPE-5). (e) HPLC coupled to light scattering detection analysis of cells treated as in (d). Bars represent mean ± s.d, *n* = 3 wells. (f) Kc167 cells were pre-fed with 400 μM OA overnight before the medium was replaced with serum free medium including either DMSO or 5 μM of CT1 (THI-4), CT2 (TPE-5) or CT3 (AU-6). After 4 h lipids were extracted and analyzed by TLC. (g) Electron micrographs of Drosophila Kc167 cells incubated in the absence or presence of 800 μM OA and DMSO or 5 μM of CT2 (TPE-5) for 18 h. (h) Murine AML12 hepatocytes were incubated with 5 μm of CT2 (TPE-5) and the rate of beta oxidation was quantified based on the CO2 production or the amount of acid soluble metabolites (ASM), respectively. Bars represent mean ± SEM, *n* = 4 wells. Also see Figures S5 and S6. The following significance levels are used: not significant (n.s.) *p* ≥ 0.05; *p < 0.05; **p < 0.01; ***p < 0.001. Results between two independent groups were determined by Student's *t*-test. Scale bars in (a,b) represent 50 μm, in (c) 10 μm and in (g) 5 μm.

We refined this analysis to determine if CT2 only blocks TAG deposition under circumstances when cells are challenged by high amounts of exogenous free fatty acids or also at physiological levels. For this purpose, we treated different models of adipocyte differentiation with CT2 in the absence of OA. Control 3T3-L1 cells (Fig. 5a), human mesenchymal stem cells (Figure S5a–c) and human visceral pre-adipocytes (Fig. 5b) showed prominent lipid stores post differentiation. However, when the cells were treated with CT2 prior to and throughout differentiation, cells did not show these lipid deposits (Fig. 5a,b and Figure S5a–c, *p*-value in Figure S5c = 0.001).

Ectopic fat deposition is a prominent complication of metabolic disorders such as obesity or type 2 diabetes and is highly associated with insulin resistance (Sattar and Gill, 2014, Shulman, 2014). Thus, we also explored the response of patient derived cells to CT2 treatment. Particularly, we tested for the ability of CT2 to block lipid deposition in differentiated myotubes obtained from a muscle biopsy of a male type 2 diabetes patient. Differentiated myotubes store LDs when they are provided with OA in the presence of DMSO or an inactive CT2 variant (Figure S5d). In contrast, when we treated with active CT2, we observed strong blockage of lipid deposition (Figure S5d).

The inability to generate lipid deposits following CT2 treatment appears to be limited to neutral lipid LDs as cholesterylester droplet deposition was unaffected (Fig. 5c and Figure S6). This is also consistent with a specific effect of CT2 on TAG synthesis. We confirmed this hypothesis by lipid profiling (by TLC or HPLC coupled to light scattering) of CT2 treated fly Kc167 or mammalian COS7 cell extracts (Fig. 5d,e; *p*-value = 8.32E-6 for TAG, *p*-value = 0.37 for PC and Figure S6b; *p*-value = 3.57E-6 for TAG, *p*-value = 0.21 for PC). These data indicate that CT2 specifically inhibits the formation of TAG-specific LDs.

While the lipid storage data are consistent with a block in the conversion of DAG to TAG, we did not observe an increase in DAG levels in TLC analyses of lipid extracts from cells treated with CT2 in the presence of OA for 16 h (Fig. 5d,e). A possible explanation is that cells divert DAG when there is no LD available that can serve as a “sink”. In support of this hypothesis, mice mutant for the DAG specific hormone-sensitive lipase accumulate DAG (Haemmerle et al., 2002). In these mice, lipogenesis is not affected and LDs are present to store DAG. In contrast, mice mutant for the enzymes converting DAG to TAG (diaclyglycerol acyltransferases 1 and 2; DGAT1 and DGAT2) are lean and do not accumulate DAG (Harris et al., 2011, Liu et al., 2011, Smith et al., 2000, Stone et al., 2004). To test the hypothesis that DAG accumulation depends on the availability of a TAG “sink”, we pre-incubated Kc167 cells with OA and combined the subsequent CT2 treatment with mild lipolytic conditions before we analyzed lipid extracts of these cells by TLC (Fig. 5f). Intriguingly, this experimental regimen did indeed result in the accumulation of 1.3-DAG specifically in combination with CT2 treatment. Absence of DAG accumulation for the other two CTs further supports different mechanisms-of-action between CT2 versus CT1 & 3.

While we focused on the role of CT2 in TAG metabolism, we also looked for evidence of adverse effects on cell function because excess OA can be toxic. The inability to store internalized free fatty acids in TAG was not toxic in our CT treatments (Fig. 1c,d) and did not result in overt ultrastructural changes to cells as determined by electron microscopy (Fig. 5g). The RNA-Seq experiments demonstrating upregulation of genes encoding beta-oxidation enzymes is consistent with the increased elimination of excess fatty acids in CT2 treated cells. Indeed, we directly observed increased fatty acid beta oxidation rates in murine AML12 cells treated with CT2 in the presence of OA (Fig. 5h, *p*-value = 1.32E-5 for CO_2_ production; *p*-value = 1.25E-4 for amount of ASM).

### 3.5 CT2 Rescues Diet and Genetically Induced Obesity in Whole Flies

**Fig. 6:**
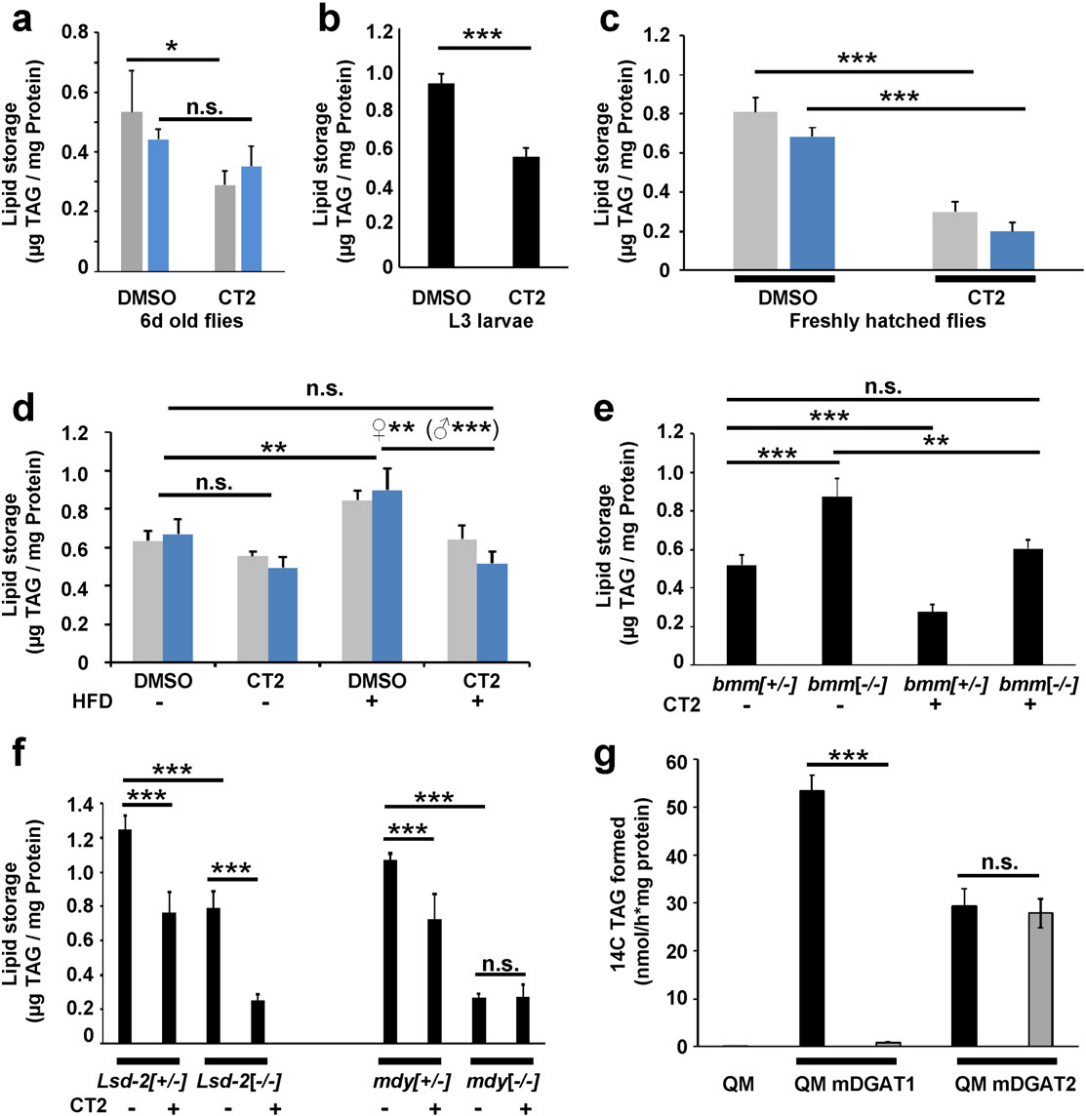
CT2 shows in vivo activity and is an inhibitor of DGAT1 activity. (a) Freshly eclosed white minus flies were transferred to food containing 10 μM CT2 (TPE-5). After 6 days of incubation at 18 °C the TAG levels were measured. (b) TAG levels of third instar larvae or (c) adult flies raised on food containing 10 μM CT2 (TPE-5). (d) TAG levels of DMSO or CT2 (TPE-5) treated flies raised on a standard or high-fat diet (HFD; 10% coconut oil) food. Flies were raised on standard food containing either DMSO or 10 μM CT2 (TPE-5), before they were transferred to standard or HFD food containing either DMSO or 10 μM CT2 (TPE-5). TAG levels were measured with 7 day old flies. (e) TAG levels of adult female flies heterozygous or homozygous for *brummer* raised on food containing either DMSO alone or 10 μM CT2 (TPE-5). (f) In vivo epistasis experiments. *Lsd-2* homo- or heterozygous and *midway* homo- or heterozygous flies were raised on fly food containing either DMSO or 10 μM of CT2 (TPE-5). Subsequently, the TAG content of freshly eclosed male flies was measured by a colorimetric assay. Colored bar plots in a, c, and d represent data for female (grey) or male (blue) adult flies. Bars in (a–f) represent mean ± s.d., n = at least three times five larvae or eight adult flies. See also Figure S7. (g) Diacylglycerol activity assays using 5 μM of TPE-5. Murine DGAT1 or DGAT2 was expressed in quadruple mutant *S.cerevisiae* cells (QM) which store no lipid (see Supplemental Experimental Procedures section for details). Bars represent mean ± s.d., *n* = 3 wells. Multiple comparisons in (d–f) were determined by one-way ANOVA followed by Bonferroni's *post-hoc* testing. All other pairwise comparisons were determined by Student's *t*-test. The following significance levels are used: not significant (n.s.) *p* ≥ 0.05; *p < 0.05; **p < 0.01; ***p < 0.001.

Small molecules active in a cellular context are not necessarily active in the whole organism. Flies can be rapidly tested for small molecule activity in physiological conditions. Freshly eclosed sex-separated adult flies which we placed for six days on CT2 containing food showed a modest reduction of TAG levels (Fig. 6a; *p*-value = 0.015 for female and 0.055 for male flies). More impressively, third instar larvae, which store copious amounts of lipid to support metamorphosis (Fig. 6b; *p*-value = 2.96E-5), as well as virgin flies (Fig. 6c; *p*-value = 1.67E-4 for female and 4.95E-6 for male flies), that completed their whole development on small molecule containing food, showed dramatically decreased TAG storage (reduction to 60% or 33% of the TAG amount of DMSO treated reference animals for larvae or freshly hatched flies, respectively). These data indicate that CT2 is bioavailable and effective in whole organisms. Flies that completed their whole development on CT2 containing food did not show any signs of toxicity, developmental defects or developmental delay indicating a general lack of adverse outcomes following CT2 treatment (data not shown).

In order to test whether CT2 might also protect from diet-induced or genetically-induced obesity, we kept small molecule treated flies on a high-fat diet (HFD) or treated flies deficient for the *brummer* lipase (Grönke et al., 2005), the fly homolog of the mammalian adipose triglyceride lipase (ATGL; (Haemmerle et al., 2006)), with CT2. Flies fed a HFD had significantly increased TAG content (Fig. 6d; *p*-value = 6.04E-3 for female and 4.00E-3 for male flies). Importantly, when flies on a HFD were treated with CT2, TAG storage amounts were greatly reduced and approached the TAG levels of control flies (Fig. 6d; *p*-value = 1 for female and 0.196 for male flies). In some cases, CT2 flies on a HFD were even leaner than those on a normal diet.

Obese *brummer* mutant flies were also more lean following CT2 treatment (Fig. 6e; *p*-value = 0.002 for female flies). Rescue of the *brummer* obesity phenotype is consistent with the idea that CT2 activity is independent of increased lipolysis levels as suggested by the tissue culture experiments shown in Fig. 4. Thus, both dietary and genetically induced obesity in flies was rescued by CT2 treatment.

### 3.6 The Activity of CT2 Stems on DGAT1 Inhibition Based on Drosophila Epistasis Experiments and a Direct Functional Assay

In order to determine if the TAG synthesis pathway was exclusively affected by CT2 in whole flies, we combined small molecule treatment with flies carrying mutations resulting in a lean phenotype. Such classical epistasis experiments have the potential to reveal whether two perturbations resulting in the same phenotype act in the same or separate pathways. In the case where the combined genetic and CT2 perturbations show an additive or synergistic effect, it is likely that the two perturbations act in separate pathways. In contrast, if there is no enhancement of the separate genetic and CT2 perturbations, they presumably act in the same pathway. As a control, we treated lean PLIN2 mutant flies, whose phenotype is based on a de-protection of LDs from lipolysis (Grönke et al., 2003) with CT2. As expected, this combination resulted in a clear additive phenotype that made flies even leaner (Fig. 6f; *p*-value = 2.2E-7). This observation strongly suggests independence of the genetic and CT2 perturbations. However, when we treated flies mutant for *midway*, which encodes the fly homolog of DGAT1 (Buszczak et al., 2002), with CT2 the phenotype was the same as observed with the individual perturbations (Fig. 6f; *p*-value = 1). Thus CT2 phenocopies *midway*. This along with the finding that *midway* mutant flies are non-responsive to CT2 strongly indicates that CT2 targets the DGAT1 TAG synthetic pathway. We found no evidence that any aspect of the CT2 phenotype is in a non-DGAT1 pathway.

We have accumulated a great deal of evidence that CT2 targets DGAT1 with high specificity. To directly test this hypothesis, we performed functional activity tests in the form of an in vitro esterification assay (Fig. 6g and Supplemental Experimental Procedures section in the online version at http://dx.doi.org/10.1016/j.ebiom.2016.04.014). For this purpose, we incubated cell extracts of a yeast quadruple mutant that is unable to synthesize neutral lipids, or extracts from such quadruple mutant cells that expressed the murine DGAT1 or DGAT2 enzyme either of which is sufficient to restore esterification activity, with radiolabeled acyl-CoA and diacylglycerol. Afterwards, we measured the amount of esterified triacylglycerol by scintillation counting. Here, we found a specific CT2 mediated inhibition of the DGAT1 enzyme activity (Fig. 6g; *p*-value = 9.30E-6 for DGAT1 and *p*-value = 0.62 for DGAT2) with an IC50 of 27 nM (Figure S7a).

## 4 Discussion

Our phenotypic screen identified a significant number of small molecules that reduce cellular lipid storage with little to no toxicity and we explored three of them in greater detail. The quantitative screening strategy, which results in dose-response information in combination with the secondary screening for cytotoxicity, provided good confidence with respect to activity. Thus, our dataset represents a valuable resource to the research community and likely harbors further hit structures worth investigation.

The RNA-Seq experiments with small molecule treated cells were able to reveal diagnostic gene expression profiles supporting different mechanisms-of-action for CTs 1/3 and 2. Indeed, further experiments such as the small molecule treatment coupled to TLC analysis of lipid extracts (Fig. 5f) supports different mechanisms. These results further consolidate the use of transcriptional profiling experiments to characterize small molecule activities in order to identify potential mechanisms-of-action for detailed follow-up (Anandhakumar et al., 2015).

Our case-study in small molecule characterization, where direct screening was done in Drosophila cells to easily segue into the facile genetics of the streamlined fly genome, resulted in the identification of a class of inhibitors of the mammalian drug target DGAT1. This enzyme was previously identified as a priority drug target, as DGAT1 mutant mice are viable and show numerous beneficial metabolic characteristics such as resistance to diet-induced obesity and increased insulin or leptin sensitivity (Smith et al., 2000). In contrast, loss of DGAT2 function in mice results in severely reduced TAG levels and death shortly after birth (Harris et al., 2011, Liu et al., 2011, Smith et al., 2000, Stone et al., 2004). These effects could not be rescued by DGAT1, which suggests fundamentally different roles of the two enzymes in mammalian lipid metabolism (Stone et al., 2004). DGAT2 variants were also detected in multiple human GWAS studies, for example associated with HDL-cholesterol levels (Willer et al., 2013), or N6 polyunsaturated fatty acid levels (Guan et al., 2014). Interestingly, no human GWAS associations are described for DGAT1, although a recent report described DGAT1 sequence variants associated with improved unsaturated milk fat concentrations in cattle (Lehnert et al., 2015).

Several lead structures for DGAT1 inhibitors have been developed, some of which entered clinical trials (Birch et al., 2010, Devita and Pinto, 2013, Matsuda and Tomoda, 2007). The CT2 TPE scaffold we identified is structurally different from the previously described DGAT1 inhibitors (Figure S7b). In 3T3L1 cells (Figure S7c), or flies (Figure S7d), CT2 outperformed two previously described DGAT1 inhibitor structures. These findings together with the activity of CT2 in cellular systems relevant to human physiology, such as the isolated human muscle cells (Figure S5d) or differentiated human visceral pre-adipocytes (Fig. 5b), further support useful applications of this CT.

While DGAT1 knockout cells are still capable of lipid deposition in the course of adipocyte differentiation (Harris et al., 2011), CT2 treatment blocked lipid deposition prominently in different systems of adipocyte differentiation. One possible explanation for this observation is that our CT2 structures also affect other cellular targets which thus results in polypharmacology. At least in flies, however, epistasis does not support the polypharmacology model (Fig. 6f and Figure S7d; *p*-value = 1). An alternative explanation for this discrepancy can be found in the varying expression levels of DGAT enzymes in different tissues. However, for Aurora kinases, or members of the phosphatidylinositol-3-OH kinase (PI(3)K) family (reviewed e.g. in: (Weiss et al., 2007)), the observed discrepancy between small molecule based inhibition and the lack-of the target protein as a result of RNAi or mutation was not based on altered expression levels of surrogate proteins. In theory, the observed differences could also be linked to the distinct roles of DGAT1 and DGAT2, which are not yet elucidated completely. In fact, ample data support functions that are only partially overlapping. For example, while DGAT2 appears to be involved in the majority of TAG synthesis in mice (Stone et al., 2004), DGAT1 knockout mice show milder phenotypes (Smith et al., 2000). However, altered DGAT1 levels affect the majority of cellular LDs (Wilfling et al., 2013). The latter finding might indicate that DGAT1 is important for the first steps of LD biogenesis. Our data potentially indicate that there is a difference between pharmacological inhibition of DGAT1 and absence of DGAT1 based on mutation. While DGAT1 is inactivated as a result of the compound activity, its presence could hinder DGAT2 to functionally replace it perhaps based on steric hindrance or preferential binding of other members of the lipogenic machinery. Thus, further experiments comparing the small molecule effects to the true loss-of-function results might help to elucidate the interplay of DGAT1 and DGAT2.

Impaired esterification of free fatty acids and acyl-CoAs due to inactivation of DGAT1 is problematic to cells. Accumulation of these metabolites has profound consequences on cells ranging from an impact on cellular signaling (Cai et al., 2011, Takahashi et al., 2006, Wellen et al., 2009) to the dysfunction and death of the cell caused by lipotoxicity (Zámbó et al., 2013, Listenberger et al., 2003, Rial et al., 2010). One way to counteract the accumulation of free fatty acids and acyl-CoAs is their beta-oxidation mediated break down. DGAT1 deficient murine primary hepatocytes are indeed protected from steatosis by decreased lipogenesis and increased beta-oxidation rates (Villanueva et al., 2009). Similarly, pharmacological inhibition of DGAT1 in enterocytes of rats enhances intestinal fatty acid oxidation and thus reduces energy intake (Schober et al., 2013). We also observed an increased expression of beta-oxidation associated genes (Fig. 3) and the beta-oxidation rate itself (Fig. 5h) downstream of CT2 treatment.

Our epistasis experiments highlighted DGAT1 as potential target of CT2 activity. This is a general advantage of testing small molecules in flies, as knockout and knockdown mutants are available for almost all genes. Additionally, most fly genes are conserved in humans, and the fly genome has not undergone the duplications of genes that occurred in the vertebrates which simplifies genetics and the evaluation of expression profiles. The fly is thus an excellent pharmacogenomics model system for tackling the problem of identifying drug targets following high-throughput phenotypic screening. As in our case, most exploratory screening campaigns are performed using tissue culture cells. However, cellular systems are limited by the absence of physiological context for testing efficacy, bioavailability, and toxicology. We see the fly as a valuable intermediary test step - with the benefit of speed, reduced ethics limitations, and a rich genetics toolbox — prior to transferring candidate small molecules to vertebrate model systems.

The following are the supplementary data related to this article.

Fig. S1, related to Fig. 1: Quantification of lipid storage amounts in fly cells and the activity of CT1 (THI-4), CT2 (TPE-5) and CT3 (AU-6) in Drosophila Kc167 cells.
Drosophila S3 (a, b) or Kc167 (a,c) cells were incubated with increasing amounts of OA for 16 h before they were fixed and stained for nuclei/DNA using Hoechst (shown in blue) and LDs by BODIPY493/503 (shown in green). Images were recorded using 20 × magnification and an ImageXpress HCS system (Molecular Devices). Images were analyzed by a custom CellProfiler image segmentation procedure and lipid storage amounts quantified as area of the detected LDs normalized by the area of the DNA/Nuclei. Bars in (b,c) represent mean ± s.d., *n* = 3 wells. (d) Cells were treated with different concentrations of the respective chemotype as described in Fig. 1d. Subsequently, the cells were processed as described in panel (a), and dose-response curves were plotted (e) as well as the number of cells quantified (f). Data in (e,f) represent mean values of three wells per data point. Scale bars represent 50 μm.
Fig. S2, related to Fig. 2: CT activity is evolutionary conserved.
Structural derivatives of CTs 1 to 3 were tested in Drosophila S3, monkey COS7 and murine AML12 cells for their ability to block lipid deposition. Structure information can be retrieved from the PubChem database (https://pubchem.ncbi.nlm.nih.gov/) using the NCGC IDs (column “CODE”). Cells were treated over night with 5 μM of the respective compound in the presence of 400 μM (S3 and COS7 cells) or 200 μM (AML12 cells) OA, fixed and stained for nuclei and LDs. Images were subsequently recorded, visually inspected and a potential LD phenotype was classified. “N” stands for no change compared to control; “N/A” stands for not analyzed; “reduced” stands for reduced lipid storage levels and “absent” for complete lack of lipid storage levels based on visual inspection of the microscopic images.
Fig. S3, related to Fig. 3: Quality control of RNA-Seq data.
(a) A histogram of normalized expression values in the logarithm scale (log (FPKM + 1)). The y-axis represents the relative number of genes/intergenic regions within a particular range of expression values (density). The vertical red line represents the 95 percentile of FPKM values for intergenic regions (FPKM 1.04 is used as a cutoff). (b) Replicate correlation matrix including all data. For each sample (e.g. “inactive_CT3”) several replicates were present and used for the clustering. For number of replicates per sample please refer to the materials & methods section. The heatmap color scale is shown on the right and corresponds to Spearman's correlation coefficient. (c) Replicate correlation matrix including data with Spearman's coefficient (rho) > 0.95. The heatmap color scale corresponds to Spearman's correlation coefficient and is recalculated for the data set with Spearman's coefficient (rho) > 0.95. (d) Inter sample correlation matrix. The heatmap color scale is the same as in panel b.
Fig. S4, related to Fig. 3: Analysis of differentially expressed (DE) genes.
(a) Boxplots representing the distribution of DE genes identified in active chemotypes (CT1 = THI-4, CT2 = TPE-69, CT3 = AU-6) vs. DMSO control or inactive chemotypes (CT1 = THI-68, CT2 = TPE-67, CT3 = AU-73) vs. DMSO control. *** — indicates *p*-value < 0.001 in Wilcoxon signed rank test. The boxplot shows that there are significantly more genes changing their expression in response to active chemotypes than to inactive ones. The number of DE genes for each samples pair is: active_CT1/DMSO_OA — 592, active_CT2/DMSO_OA — 171, active_CT3/DMSO_OA — 284, inactive_CT1/DMSO_OA — 47, inactive_CT2/DMSO_OA — 105, inactive_CT3/DMSO_OA — 49, DMSO_noOA/DMSO_OA — 436. The detailed information is provided in Table S3. (b) Boxplots representing the distribution of DE genes identified in active chemotypes vs. DMSO control, inactive chemotypes vs. DMSO control, and DE genes that are common for both active and inactive vs. DMSO control along with their direction of change (yellow = increased expression, blue = decreased expression). These boxplots show that the number of DE genes changing in active chemotypes vs. DMSO control is higher than in the other two conditions indicating specificity of DE gene response in active chemotypes. (c) A heatmap of Modulated Modularity Clustering of 15 different functional modules of DE genes identified in the comparison “active chemotypes vs. DMSO”. Black numbers in the heatmap indicate modules. Color code corresponds to Pearson's (r) correlation coefficient, with the heatmap scale shown on the right. Gene lists from each module are presented in Table S4. (d) GO terms enriched for genes belonging to the clusters 1, 3, 4 or 5 shown on the heatmap in Fig. 3a. There was no significant enrichment of GO-terms for cluster 2. For details see Table S5. (e) Gene Ontology (GO) terms enriched for DE genes identified in each active chemotype (CT1, CT2, and CT3) vs. DMSO. Analysis was done using the GOrilla software based on the exact minimum hypergeometric (mHG) *p*-value algorithm and the REViGO program to filter secondary GO terms.
Fig. S5, related to Fig. 5: CT2 (TPE-5) blocks lipid deposition during the differentiation of human stem cells and in differentiated human myotubes.
(a) hMSCs deposit large amounts of lipids during differentiation. If CT2 (TPE-5) is added pre-induction, or at the different induction rounds, this lipid deposition is efficiently blocked. Green: LDs (BODIPY493/503), blue: DNA/nuclei (DAPI). Scale bar represents 30 μm. (b) CT2 (TPE-5) blocks lipid deposition in differentiating hMSCs derived of the Mid-Atlantic Nutrition Obesity Research Center (NORC). hMSCs deposit large amounts of lipids during the differentiation as revealed by bright field images or OilRedO staining quantification (c). Treatment of the cells with 1 μM CT2 (TPE-5) during the differentiation process resulted in a block of lipid deposition. Images were recorded with a standard tissue culture microscope with a 10 × objective. Bars represent mean ± SEM, *n* = 2 wells. Pairwise comparison statistics was determined by Student's *t*-test (*p*-value = 0.0014). (d) Differentiated myotubes obtained from a muscle biopsy of a male type 2 diabetes patient deposit LDs when they are provided with 100 μM OA in the presence of DMSO only, or 5 μM of an inactive CT2 structure (TPE-67). When 5 μM of the active CT2 structure TPE-5 were present, however, lipid deposition was prominently blocked. Cells were stained with DAPI for DNA/nuclei and BODIPY493/503 for LDs. Scale bar represents 1 μm.
Fig. S6, related to Fig. 5: CT2 does not block cholesterylester droplet deposition in AML12 cells and causes a TAG-specific lipid storage phenotype in COS-7 cells.
(a) AML12 cells were incubated with NBD-cholesterol (shown in green) in the absence or presence of OA and DMSO or 5 μM CT2 (TPE-5) for 18 h. Subsequently, the cells were fixed, stained for DNA/nuclei (Hoechst33342 shown in blue) and LDs (LipidTOX HCS Deep Red, shown in red) and analyzed by microscopy. Scale bar represents 50 μm. (b) Thin layer chromatography of COS7 cell lipid extracts. The cells were treated either with DMSO only or with 5 μM CT2 (TPE-5) and lipid deposition was induced by providing radiolabeled OA. (c) Lipid extracts of COS7 cells treated with the DMSO solvent only or 5 μM CT2 (TPE-5) were analyzed by HPLC coupled to light scattering. The cells were loaded with cold OA. Bars represent mean ± s.d., *n* = 3 wells. *p*-value = 3.57E-6 for TAG and *p*-value = 0.21 for PC estimated by a Student's *t*-test.
Fig. S7, related to Fig. 6: Dose-response curve of CT2 (TPE-5), overview of published DGAT1 small molecule inhibitors, and comparison between CT2 activity and exemplary DGAT1 inhibitors in cells and in vivo.
(a) CT2 (TPE-5) inhibits activity of murine DGAT1 expressed in a quadruple yeast mutant which is unable to deposit lipid stores. Experiments were performed as described in Fig. 6G. Data is represented as mean ± s.d., *n* = 3 wells. (b) Published DGAT1 inhibitor structures include: (1) early benzazepinedione compound (Merck) (Liu et al., 2013), (2) T863 (Japan Tobacco/Tularik and Pfizer) (Cao et al., 2011, Fox et al., 2014), (3) PF-04620110 (Pfizer) (Dow et al., 2011), (4) AZD7687 (Astra Zeneca) (McCoull et al., 2012), (5) LCQ908 (Novartis) (Serrano-Wu et al., 2012), (6) A-922500 (Abbott) (Zhao et al., 2008), (7) Compound-14 (Abbott) (Yeh et al., 2012), (8) Compound-A (Takeda pharmaceutical Company Ltd.) (Yamamoto et al., 2011). (c) Dose-response curve for the inhibition of OA-induced lipid storage levels in response to A-922500 or CT2 (TPE-5) in 3T3-L1 cells incubated over night with 400 μM OA. Data represent single well measurements. (d) TAG levels of third instar larvae, or sex-separated non-mated 1 d old adult flies which were raised on fly food supplemented with PF04620110 or CT2 (TPE-5) alone or in combination (fly experiments were performed as described in Fig. 6c). Bars show mean ± s.d., *n* = 4 times 5 larvae, or 8 flies, respectively. Multiple comparisons were determined by one-way ANOVA followed by Bonferroni's *post-hoc* testing. Image 1

Table S1 RNA-Seq gene expression values (FPKM values) for each separate sample. Table S1

Table S2 RNA-Seq gene expression values (FPKM values) per sample. Table S2

Table S3 CuffDiff results indicating significance of differential gene expression between two samples. Table S3

Table S4 The table summarizes the results for 919 differentially expressed genes used for the clusters shown in Fig. 3a and Fig. S4c. Table S4

Table S5 Details of the GO-class enrichment of the clusters shown in Fig. 3a. Table relates also to Figure S4d. Table S5

Supplemental Experimental Procedures. Image 2

## Funding sources

This work was in part financed by the Max Planck Society, the Strategic Research Funds of the University of Duesseldorf (F2012/279-6; M. Beller), the German Federal Ministry of Education and Research (BMBF; 031A306; M. Beller), the Austrian Science Fund (FWF; P25193; A. Lass), the University of Maryland (Geriatric Research, Education and Clinical Center (GRECC); Clinical Nutrition Research Unit (P30 DK072488)), the Baltimore Veterans Affairs Health Care Center, and the National Institutes of Health (intramural: NIH/NIDDK/NCATS; extramural: 1Ro3 MH085686-0; M.Beller). The funders had no role in the design of the study, the writing of the manuscript, or the decision to submit it for publication.

## Conflict of interest statement

We declare that this study does not have financial or other relationships that might lead to a conflict of interest.

## Author contributions

KT, YQZ, LL, KG, TS, TOE, MLN, US, JML, CS, DA, ZL, MS, MBB, and MB performed experiments, collected data, analyzed data and prepared figures.

SZ, CS, AL, DA, BO, HW, ZL, MS, MBB, and MB supervised and planned experiments.

KT, TOE, CS, AL, DA, BO, HW, ZL, MS, MBB, and MB wrote the manuscript.
