# Supplementary material for: A Class of Diacylglycerol Acyltransferase 1 Inhibitors Identified by a Combination of Phenotypic High-throughput Screening, Genomics, and Genetics
Source: eBioMedicine. 2016 Apr 16;8:49–59. doi: 10.1016/j.ebiom.2016.04.014 (PMC4919474; doi:10.1016/j.ebiom.2016.04.014)
Supplement: Supplementary file 1 — Fig. S1, related to Fig. 1: Quantification of lipid storage amounts in fly cells and the activity of CT1 (THI-4), CT2 (TPE-5) and CT3 (AU-6) in Drosophila Kc167 cells. Drosophila S3 (a, b) or Kc167 (a,c) cells were incubated with increasing amounts of OA for 16 h before they were fixed and stained for nuclei/DNA using Hoechst (shown in blue) and LDs by BODIPY493/503 (shown in green). Images were recorded using 20 × magnification and an ImageXpress HCS system (Molecular Devices). Images were analyzed by a custom CellProfiler image segmentation procedure and lipid storage amounts quantified as area of the detected LDs normalized by the area of the DNA/Nuclei. Bars in (b,c) represent mean ± s.d., n = 3 wells. (d) Cells were treated with different concentrations of the respective chemotype as described in Fig. 1d. Subsequently, the cells were processed as described in panel (a), and dose-response curves were plotted (e) as well as the number of cells quantified (f). Data in (e,f) represent mean values of three wells per data point. Scale bars represent 50 μm. Fig. S2, related to Fig. 2: CT activity is evolutionary conserved. Structural derivatives of CTs 1 to 3 were tested in Drosophila S3, monkey COS7 and murine AML12 cells for their ability to block lipid deposition. Structure information can be retrieved from the PubChem database (https://pubchem.ncbi.nlm.nih.gov/) using the NCGC IDs (column “CODE”). Cells were treated over night with 5 μM of the respective compound in the presence of 400 μM (S3 and COS7 cells) or 200 μM (AML12 cells) OA, fixed and stained for nuclei and LDs. Images were subsequently recorded, visually inspected and a potential LD phenotype was classified. “N” stands for no change compared to control; “N/A” stands for not analyzed; “reduced” stands for reduced lipid storage levels and “absent” for complete lack of lipid storage levels based on visual inspection of the microscopic images. Fig. S3, related to Fig. 3: Quality control of RNA-Seq data. (a [file mmc1.pdf]

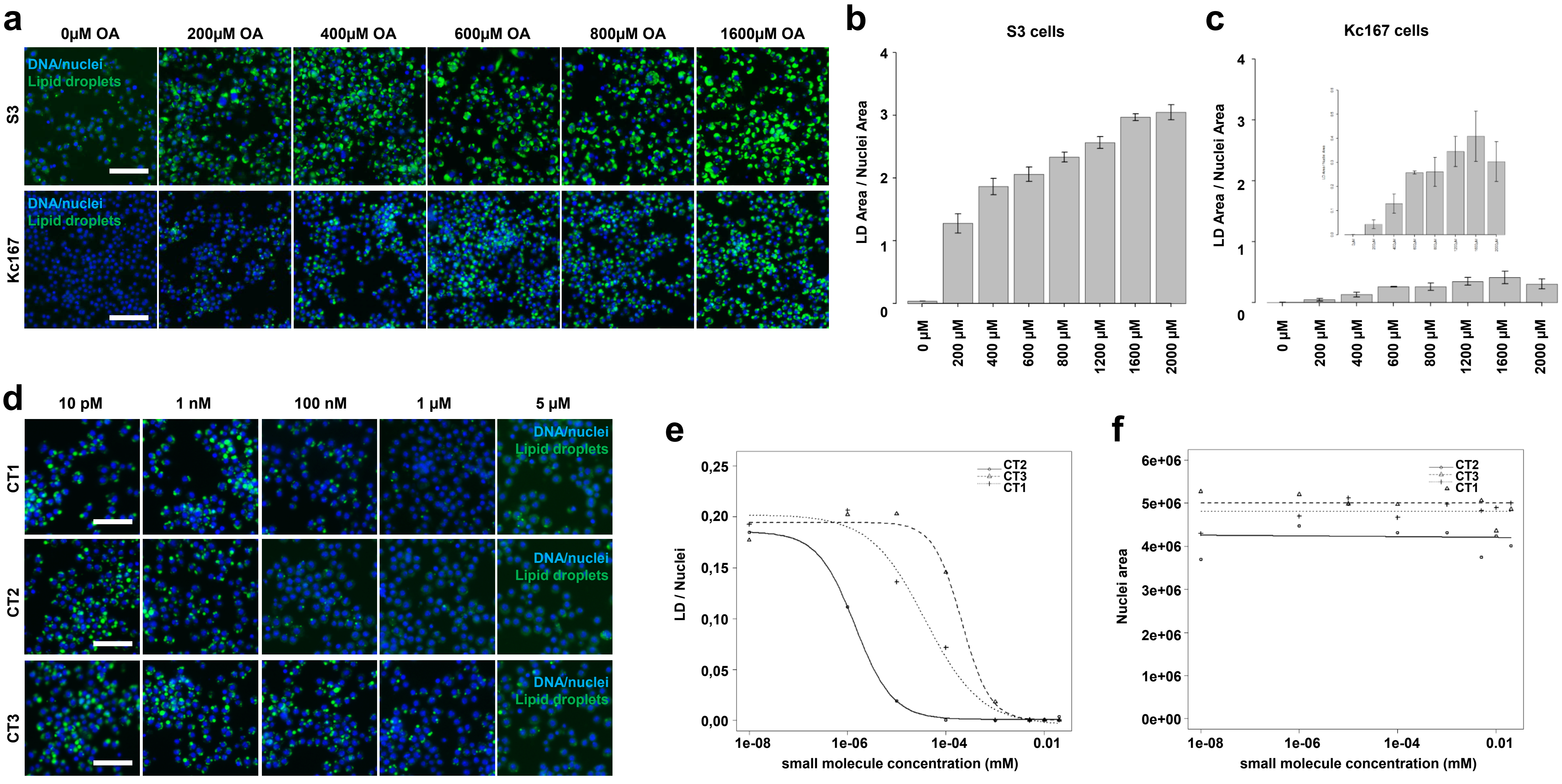

a
0µM OA
200µM OA
400µM OA
600µM OA
800µM OA
1600µM OA
DNA/nuclei
Lipid droplets
S3
Kc167
b
S3 cells
LD Area / Nuclei Area
0 µM
200 µM
400 µM
600 µM
800 µM
1200 µM
1600 µM
2000 µM
c
Kc167 cells
LD Area / Nuclei Area
d
10 pM
1 nM
100 nM
1 µM
5 µM
CT1
CT2
CT3
e
LD / Nuclei
small molecule concentration (mM)
f
Nuclei area
small molecule concentration (mM)


# Figure S1

**Fig. S1**, related to Fig. 1: Quantification of lipid storage amounts in fly cells and the activity of CT1 (THI-4), CT2 (TPE-5) and CT3 (AU-6) in Drosophila Kc167 cells. Drosophila S3 (a, b) or Kc167 (a,c) cells were incubated with increasing amounts of OA for 16 h before they were fixed and stained for nuclei/DNA using Hoechst (shown in blue) and LDs by BODIPY493/503 (shown in green). Images were recorded using 20 × magnification and an ImageXpress HCS system (Molecular Devices). Images were analyzed by a custom CellProfiler image segmentation procedure and lipid storage amounts quantified as area of the detected LDs normalized by the area of the DNA/Nuclei. Bars in (b,c) represent mean ± s.d., $n$ = 3 wells. (d) Cells were treated with different concentrations of the respective chemotype as described in Fig. 1d. Subsequently, the cells were processed as described in panel (a), and dose-response curves were plotted (e) as well as the number of cells quantified (f). Data in (e,f) represent mean values of three wells per data point. Scale bars represent 50 μm.

**a**

| CODE | S3 | COS7 | AML12 | Compound # |
|---|---|---|---|---|
| MLS001090699-01 | Absent | N/A | N/A | 1 |
| NCGC00241451-01 | N | N | N | 7 |
| NCGC00242548-01 | Absent | N | Reduced | 8 |
| NCGC00242549-01 | Absent | Absent | Absent | THI-4 |
| NCGC00242551-01 | Absent | Reduced | N | 9 |
| NCGC00242572-01 | Reduced | N | N | 10 |
| NCGC00242573-01 | Absent | Absent | Reduced | 11 |
| NCGC00242574-01 | Absent | Absent | Reduced | 12 |
| NCGC00238536-01 | Reduced | N | N | 13 |
| NCGC00238537-01 | Absent | Absent | N | 14 |
| NCGC00238555-01 | N | N | N | 15 |
| NCGC00238557-01 | Reduced | N | Reduced | 16 |
| NCGC00238558-01 | N | N | N | 17 |
| NCGC00238559-01 | Absent | N | N | 18 |
| NCGC00238560-01 | N | N | N | 19 |
| NCGC00238547-01 | Reduced | N | N | 20 |
| NCGC00238548-01 | Absent | Reduced | N | 21 |
| NCGC00238544-01 | N | N | N | 22 |
| NCGC00238553-01 | Reduced | N | N | 23 |
| NCGC00238539-01 | Absent | Absent | N | 24 |
| NCGC00238541-01 | Absent | N | N | 25 |
| NCGC00238561-01 | Reduced | N | N | 26 |
| NCGC00189332-02 | Absent | Absent | N | 27 |
| NCGC00189555-01 | Absent | N | Reduced | 28 |
| NCGC00250255-01 | N | N | N | 68 |
| NCGC00189556-01 | N | N | N | 29 |

**b**

| CODE | S3 | COS7 | AML12 | Compound # |
|---|---|---|---|---|
| NCGC00241414-01 | N | N | N | 30 |
| NCGC00241418-01 | N | N | N | 31 |
| NCGC00241426-01 | Absent | Absent | Reduced | 32 |
| NCGC00241442-01 | N | N | N | 33 |
| NCGC00241415-01 | N | N | N | 34 |
| NCGC00241419-01 | N | N | Reduced | 35 |
| NCGC00241427-01 | Reduced | Absent | Reduced | 36 |
| NCGC00241443-01 | N | N | N | 37 |
| NCGC00241416-01 | N | N | Reduced | 38 |
| NCGC00241428-01 | Absent | Absent | Reduced | 39 |
| NCGC00241433-01 | N | N | N | 40 |
| NCGC00241440-01 | N | N | Reduced | 41 |
| NCGC00241420-01 | N | N | N | 42 |
| NCGC00241429-01 | Reduced | Absent | Reduced | TPE-5 |
| NCGC00241437-01 | N | N | N | 43 |
| NCGC00241441-01 | Reduced | N | N | 44 |
| NCGC00241421-01 | N | N | N | 45 |
| NCGC00241430-01 | Reduced | Absent | N | 46 |
| NCGC00241439-01 | Reduced | N | N | 47 |
| NCGC00241444-01 | N | N | N | 48 |
| NCGC00241452-01 | N | N | N | 49 |
| NCGC00241422-01 | N | N | Reduced | 50 |
| NCGC00241431-01 | Absent | Absent | R | 51 |
| NCGC00241434-01 | N | Reduced | N | 52 |
| NCGC00241445-01 | N | N | N | 53 |
| NCGC00241453-01 | N | Reduced | N | 54 |
| NCGC00241423-01 | N | Reduced | Reduced | 55 |
| NCGC00241424-01 | Absent | Absent | Reduced | 56 |
| NCGC00241435-01 | N | N | N | 57 |
| NCGC00241438-01 | N | N | N | 58 |
| NCGC00241417-01 | N | Reduced | N | 59 |
| NCGC00241425-01 | N | Absent | Reduced | 60 |
| NCGC00241436-01 | N | N | N | 61 |
| NCGC00241432-01 | N | N | N | 62 |
| NCGC00238550-01 | Reduced | Absent | N | 63 |
| NCGC00092589-02 | Reduced | Absent | N | 2 |
| NCGC00238551-01 | Absent | Absent | Reduced | 64 |
| NCGC00238546-02 | Reduced | Absent | N | 65 |
| NCGC00241007-01 | N | N | N | 66 |
| NCGC00238540-02 | N | N | N | 67 |
| NCGC00345354-01 | Absent | Absent | Absent | 69 |

**c**

| CODE | S3 | COS7 | AML12 | Compound # |
|---|---|---|---|---|
| NCGC00241448-01 | Reduced | Reduced | Reduced | 70 |
| NCGC00034917-03 | Reduced | Absent | Reduced | 3 |
| NCGC00241449-01 | N | N | N | 71 |
| NCGC00241450-01 | N | Reduced | N | 72 |
| NCGC00241446-01 | N | N | N | 73 |
| NCGC00241447-01 | Reduced | N | N | 74 |
| NCGC00242550-01 | Reduced | N | N | 75 |
| NCGC00242553-01 | Reduced | N | N | 76 |
| NCGC00242554-01 | N | Reduced | N | 77 |
| NCGC00242555-01 | Absent | Absent | N | 78 |
| NCGC00242556-01 | Absent | Absent | Reduced | 79 |
| NCGC00242557-01 | N | N | N | 80 |
| NCGC00242558-01 | N | N | N | 81 |
| NCGC00242559-01 | Reduced | N | N | 82 |
| NCGC00242560-01 | Absent | Absent | Reduced | AU-6 |
| NCGC00242563-01 | N | N | Reduced | 83 |
| NCGC00242564-01 | Reduced | N | N | 84 |
| NCGC00242565-01 | N | N | N | 85 |
| NCGC00242566-01 | Reduced | N | N | 86 |
| NCGC00242567-01 | N | N | N | 87 |
| NCGC00242568-01 | Absent | Absent | N | 88 |
| NCGC00238542-01 | Reduced | N | Reduced | 89 |

# Figure S2

**Fig. S2**, related to Fig. 2: CT activity is evolutionary conserved. Structural derivatives of CTs 1 to 3 were tested in Drosophila S3, monkey COS7 and murine AML12 cells for their ability to block lipid deposition. Structure information can be retrieved from the PubChem database (https://pubchem.ncbi.nlm.nih.gov/) using the NCGC IDs (column "CODE"). Cells were treated over night with 5 µM of the respective compound in the presence of 400 µM (S3 and COS7 cells) or 200 µM (AML12 cells) OA, fixed and stained for nuclei and LDs. Images were subsequently recorded, visually inspected and a potential LD phenotype was classified. "N" stands for no change compared to control; "N/A" stands for not analyzed; "reduced" stands for reduced lipid storage levels and "absent" for complete lack of lipid storage levels based on visual inspection of the microscopic images.

Figure S3

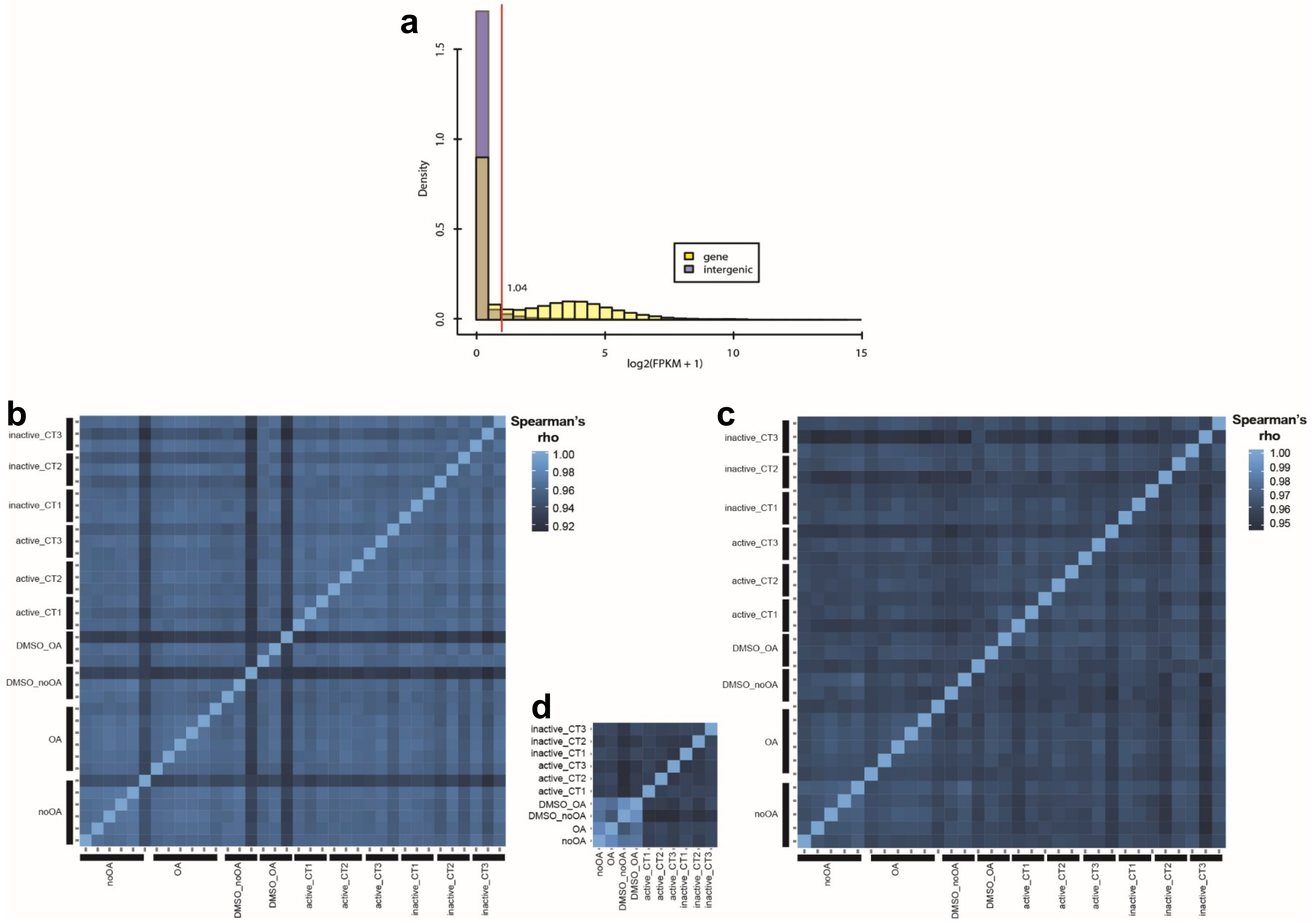

a
Density
log2(FPKM + 1)
1.04
gene
intergenic
b
Spearman's rho
1.00
0.98
0.96
0.94
0.92
inactive_CT3
inactive_CT2
inactive_CT1
active_CT3
active_CT2
active_CT1
DMSO_OA
DMSO_noOA
OA
noOA
c
Spearman's rho
1.00
0.99
0.98
0.97
0.96
0.95
d

**Fig. S3**, related to Fig. 3: Quality control of RNA-Seq data. (a) A histogram of normalized expression values in the logarithm scale (log (FPKM+1)). The y-axis represents the relative number of genes/intergenic regions within a particular range of expression values (density). The vertical red line represents the 95 percentile of FPKM values for intergenic regions (FPKM 1.04 is used as a cutoff). (b) Replicate correlation matrix including all data. For each sample (e.g. "inactive_CT3") several replicates were present and used for the clustering. For number of replicates per sample please refer to the materials & methods section. The heatmap color scale is shown on the right and corresponds to Spearman's correlation coefficient. (c) Replicate correlation matrix including data with Spearman's coefficient (rho) > 0.95. The heatmap color scale corresponds to Spearman's correlation coefficient and is recalculated for the data set with Spearman's coefficient (rho) > 0.95. (d) Inter sample correlation matrix. The heatmap color scale is the same as in panel b.

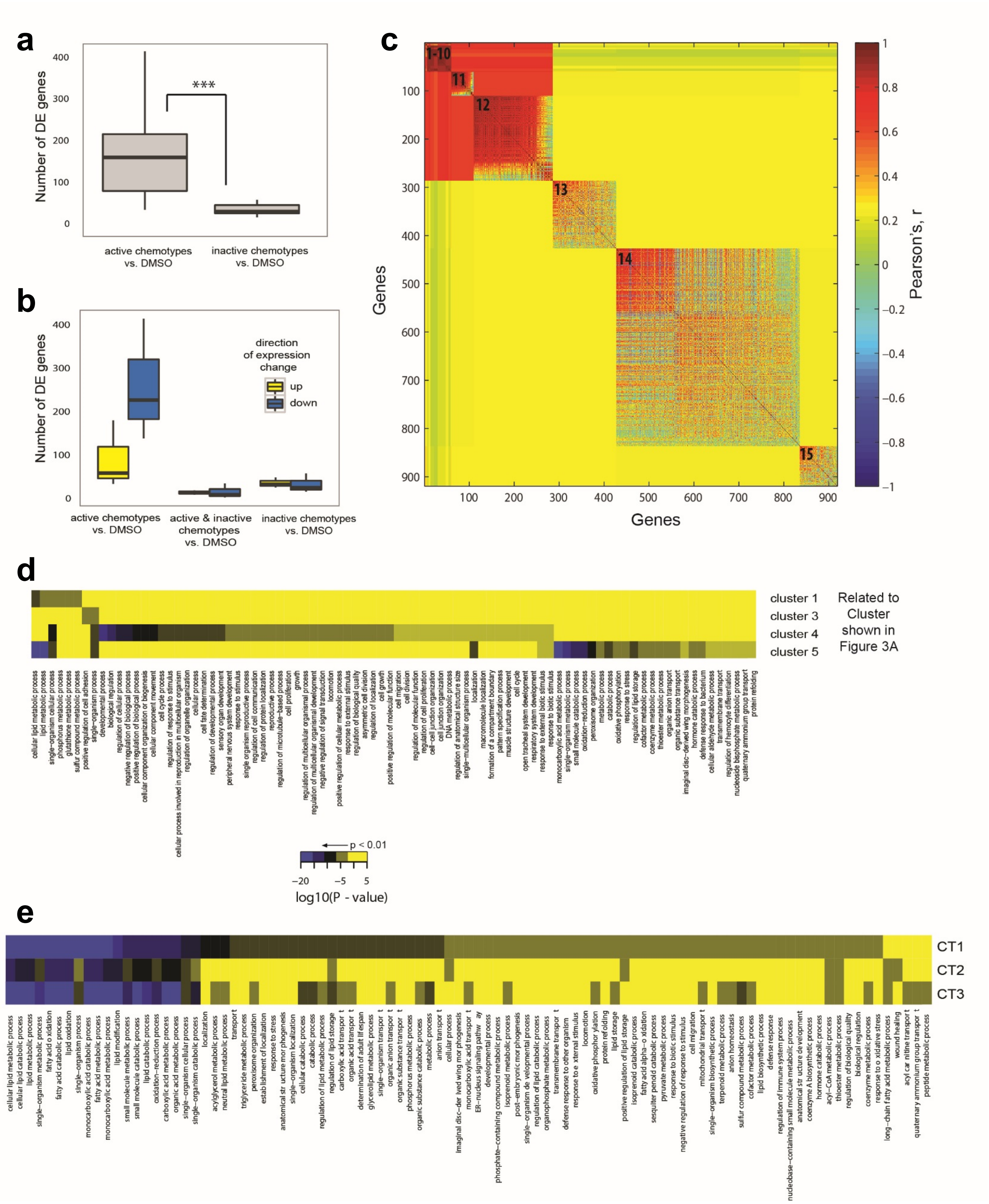
a
Number of DE genes
***
active chemotypes vs. DMSO
inactive chemotypes vs. DMSO
b
Number of DE genes
direction of expression change
up
down
active chemotypes vs. DMSO
active & inactive chemotypes vs. DMSO
inactive chemotypes vs. DMSO
c
1-10
11
12
13
14
15
Genes
Genes
Pearson's, r
d
cluster 1
cluster 3
cluster 4
cluster 5
Related to Cluster shown in Figure 3A
cellular lipid metabolic process
lipid metabolic process
single-organism cellular process
phosphorus metabolic process
glutathione metabolic process
sulfur compound metabolic process
positive regulation of cell adhesion
single-organism process
developmental process
biological regulation
regulation of cellular process
negative regulation of biological process
positive regulation of biological process
cellular component organization or biogenesis
cellular component movement
cell cycle process
regulation of response to stimulus
cellular process involved in reproduction in multicellular organism
regulation of organelle organization
cellular process
cell fate determination
regulation of developmental process
sensory organ development
peripheral nervous system development
response to stimulus
single organism reproductive process
regulation of cell communication
regulation of protein localization
reproductive process
regulation of microtubule-based process
cell proliferation
growth
regulation of multicellular organismal process
regulation of multicellular organismal development
negative regulation of signal transduction
locomotion
positive regulation of cellular metabolic process
response to external stimulus
regulation of biological quality
asymmetric cell division
regulation of localization
cell growth
positive regulation of molecular function
cell migration
cell division
regulation of molecular function
regulation of cell proliferation
cell-cell junction organization
cell junction organization
DNA metabolic process
regulation of anatomical structure size
single-multicellular organism process
localization
macromolecule localization
formation of a compartment boundary
pattern specification process
muscle structure development
cell cycle
open tracheal system development
respiratory system development
response to external biotic stimulus
response to biotic stimulus
monocarboxylic acid metabolic process
single-organism metabolic process
small molecule catabolic process
oxidation-reduction process
peroxisome organization
metabolic process
catabolic process
oxidative phosphorylation
response to stress
regulation of lipid storage
cofactor metabolic process
coenzyme metabolic process
thioester metabolic process
organic anion transport
organic substance transport
imaginal disc-derived wing morphogenesis
hormone catabolic process
defense response to bacterium
cellular aldehyde metabolic process
transmembrane transport
regulation of hemocyte differentiation
nucleoside bisphosphate metabolic process
quaternary ammonium group transport
protein refolding
p < 0.01
log10(P - value)
e
CT1
CT2
CT3
cellular lipid metabolic process
cellular lipid catabolic process
lipid metabolic process
single-organism metabolic process
fatty acid oxidation
fatty acid catabolic process
lipid oxidation
single-organism process
monocarboxylic acid catabolic process
fatty acid metabolic process
monocarboxylic acid metabolic process
lipid modification
small molecule metabolic process
small molecule catabolic process
lipid catabolic process
oxidation-reduction process
carboxylic acid metabolic process
organic acid metabolic process
single-organism cellular process
single-organism catabolic process
localization
acylglycerol metabolic process
neutral lipid metabolic process
transport
triglyceride metabolic process
peroxisome organization
establishment of localization
response to stress
anatomical structure morphogenesis
single-organism localization
cellular catabolic process
catabolic process
regulation of lipid storage
carboxylic acid transport
organic acid transport
determination of adult lifespan
glycerolipid metabolic process
single-organism transport
organic anion transport
organic substance transport
phosphorus metabolic process
organic substance catabolic process
metabolic process
anion transport
cellular process
imaginal disc-derived wing morphogenesis
monocarboxylic acid transport
ER-nucleus signaling pathway
developmental process
phosphate-containing compound metabolic process
isoprenoid metabolic process
post-embryonic morphogenesis
single-organism developmental process
regulation of lipid catabolic process
organophosphate metabolic process
transmembrane transport
defense response to other organism
response to external stimulus
locomotion
oxidative phosphorylation
protein refolding
lipid storage
positive regulation of lipid storage
isoprenoid catabolic process
fatty acid alpha-oxidation
sesquiterpenoid catabolic process
pyruvate metabolic process
response to biotic stimulus
negative regulation of response to stimulus
cell migration
mitochondrial transport
single-organism biosynthetic process
terpenoid metabolic process
anion homeostasis
sulfur compound metabolic process
cofactor metabolic process
lipid biosynthetic process
defense response
regulation of immune system process
nucleobase-containing small molecule metabolic process
anatomical structure development
coenzyme A biosynthetic process
hormone catabolic process
acyl-CoA metabolic process
thioester metabolic process
regulation of biological quality
biological regulation
coenzyme metabolic process
response to oxidative stress
long-chain fatty acid metabolic process
wound healing
acyl carnitine transport
quaternary ammonium group transport
peptide metabolic process

# Figure S4

**Fig. S4**, related to Fig. 3: Analysis of differentially expressed (DE) genes. (a) Boxplots representing the distribution of DE genes identified in active chemotypes (CT1 = THI-4, CT2 = TPE-69, CT3 = AU-6) vs. DMSO control or inactive chemotypes (CT1 = THI-68, CT2 = TPE-67, CT3 = AU-73) vs. DMSO control. *** - indicates *p*-value < 0.001 in Wilcoxon signed rank test. The boxplot shows that there are significantly more genes changing their expression in response to active chemotypes than to inactive ones. The number of DE genes for each samples pair is: active_CT1/DMSO_OA – 592, active_CT2/DMSO_OA – 171, active_CT3/DMSO_OA – 284, inactive_CT1/DMSO_OA – 47, inactive_CT2/DMSO_OA – 105, inactive_CT3/DMSO_OA – 49, DMSO_noOA/DMSO_OA – 436. The detailed information is provided in Table S3. (b) Boxplots representing the distribution of DE genes identified in active chemotypes vs. DMSO control, inactive chemotypes vs. DMSO control, and DE genes that are common for both active and inactive vs. DMSO control along with their direction of change (yellow = increased expression, blue = decreased expression). These boxplots show that the number of DE genes changing in active chemotypes vs. DMSO control is higher than in the other two conditions indicating specificity of DE gene response in active chemotypes. (c) A heatmap of Modulated Modularity Clustering of 15 different functional modules of DE genes identified in the comparison "active chemotypes vs. DMSO". Black numbers in the heatmap indicate modules. Color code corresponds to Pearson's (r) correlation coefficient, with the heatmap scale shown on the right. Gene lists from each module are presented in Table S4. (d) GO terms enriched for genes belonging to the clusters 1, 3, 4 or 5 shown on the heatmap in Fig. 3a. There was no significant enrichment of GO-terms for cluster 2. For details see Table S5. (e) Gene Ontology (GO) terms enriched for DE genes identified in each active chemotype (CT1, CT2, and CT3) vs. DMSO. Analysis was done using the GOrilla software based on the exact minimum hypergeometric (mHG) *p*-value algorithm and the REViGO program to filter secondary GO terms.

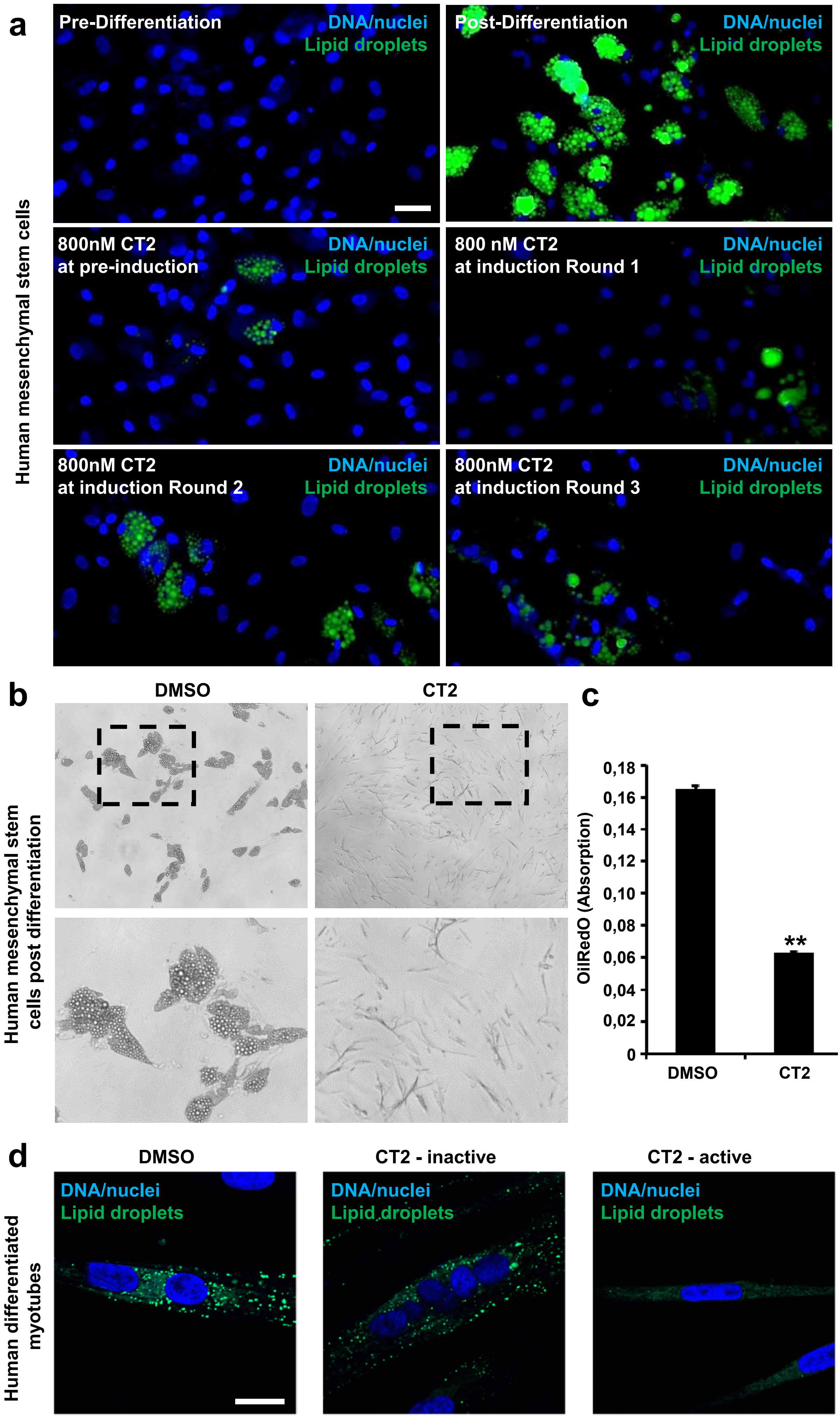
a
Pre-Differentiation
DNA/nuclei
Lipid droplets
Post-Differentiation
DNA/nuclei
Lipid droplets
800nM CT2
at pre-induction
DNA/nuclei
Lipid droplets
800 nM CT2
at induction Round 1
DNA/nuclei
Lipid droplets
800nM CT2
at induction Round 2
DNA/nuclei
Lipid droplets
800nM CT2
at induction Round 3
DNA/nuclei
Lipid droplets
Human mesenchymal stem cells
b
DMSO
CT2
Human mesenchymal stem cells post differentiation
c
OilRedO (Absorption)
0,18
0,16
0,14
0,12
0,10
0,08
0,06
0,04
0,02
0
**
DMSO
CT2
d
DMSO
CT2 - inactive
CT2 - active
DNA/nuclei
Lipid droplets
Human differentiated myotubes

Figure S5

**Fig. S5**, related to Fig. 5: CT2 (TPE-5) blocks lipid deposition during the differentiation of human stem cells and in differentiated human myotubes. (a) hMSCs deposit large amounts of lipids during differentiation. If CT2 (TPE-5) is added pre-induction, or at the different induction rounds, this lipid deposition is efficiently blocked. Green: LDs (BODIPY493/503), blue: DNA/nuclei (DAPI). Scale bar represents 30 µm. (b) CT2 (TPE-5) blocks lipid deposition in differentiating hMSCs derived of the Mid-Atlantic Nutrition Obesity Research Center (NORC). hMSCs deposit large amounts of lipids during the differentiation as revealed by bright field images or OilRedO staining quantification (c). Treatment of the cells with 1 µM CT2 (TPE-5) during the differentiation process resulted in a block of lipid deposition. Images were recorded with a standard tissue culture microscope with a 10× objective. Bars represent mean ± SEM, *n* = 2 wells. Pairwise comparison statistics was determined by Student's *t*-test (*p*-value = 0.0014). (d) Differentiated myotubes obtained from a muscle biopsy of a male type 2 diabetes patient deposit LDs when they are provided with 100 µM OA in the presence of DMSO only, or 5 µM of an inactive CT2 structure (TPE-67). When 5 µM of the active CT2 structure TPE-5 were present, however, lipid deposition was prominently blocked. Cells were stained with DAPI for DNA/nuclei and BODIPY493/503 for LDs. Scale bar represents 1 µm.

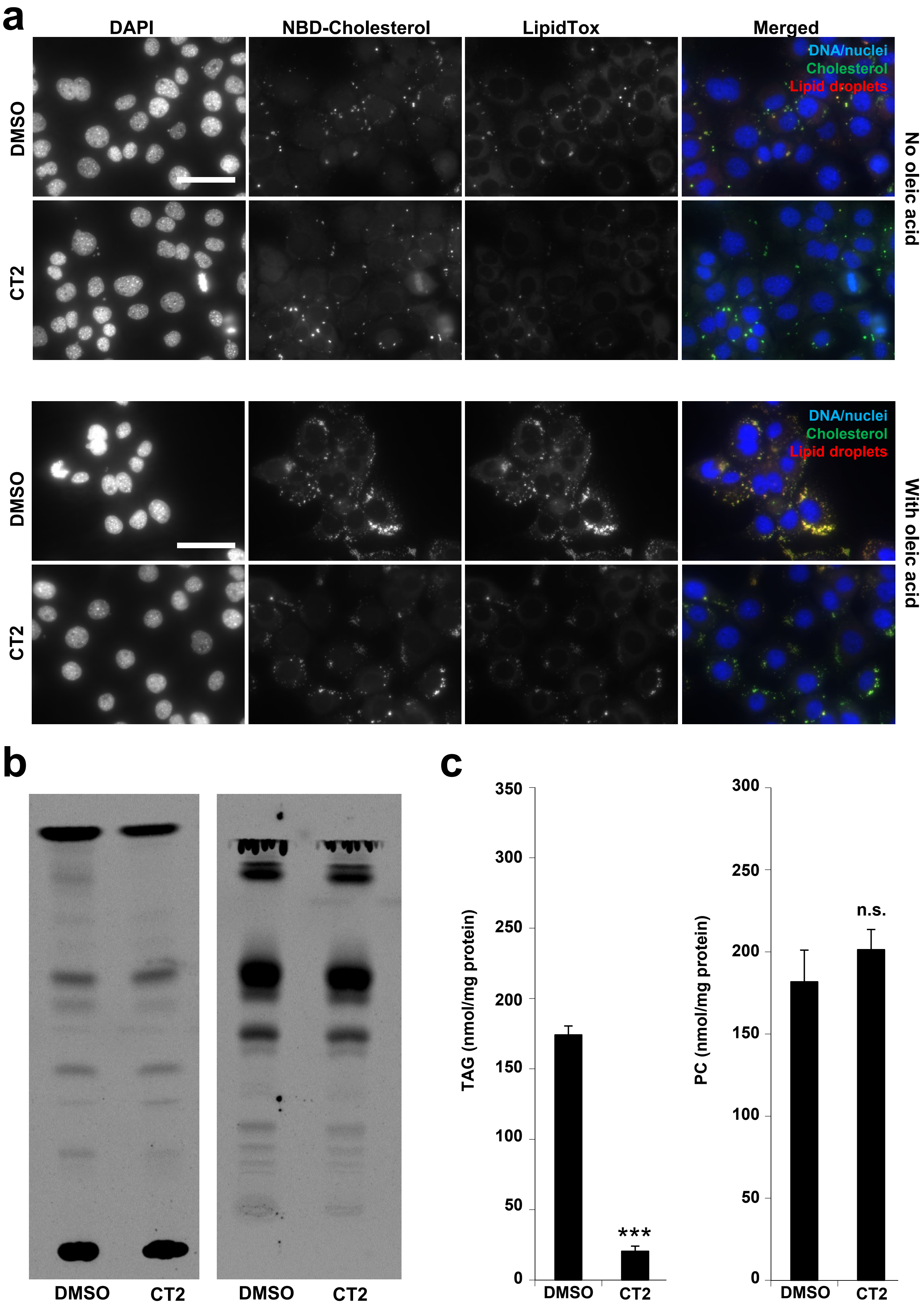
a
DAPI
NBD-Cholesterol
LipidTox
Merged
DNA/nuclei
Cholesterol
Lipid droplets
DMSO
CT2
No oleic acid
DNA/nuclei
Cholesterol
Lipid droplets
DMSO
CT2
With oleic acid
b
DMSO
CT2
DMSO
CT2
c
TAG (nmol/mg protein)
350
300
250
200
150
100
50
0
***
DMSO
CT2
PC (nmol/mg protein)
300
250
200
150
100
50
0
n.s.
DMSO
CT2

# Figure S6

**Fig. S6**, related to Fig. 5: CT2 does not block cholesterylester droplet deposition in AML12 cells and causes a TAG-specific lipid storage phenotype in COS-7 cells. (a) AML12 cells were incubated with NBD-cholesterol (shown in green) in the absence or presence of OA and DMSO or 5 µM CT2 (TPE-5) for 18 hours. Subsequently, the cells were fixed, stained for DNA/nuclei (Hoechst33342 shown in blue) and LDs (LipidTOX HCS Deep Red, shown in red) and analyzed by microscopy. Scale bar represents 50 µm. (b) Thin layer chromatography of COS7 cell lipid extracts. The cells were treated either with DMSO only or with 5 µM CT2 (TPE-5) and lipid deposition was induced by providing radiolabeled OA. (c) Lipid extracts of COS7 cells treated with the DMSO solvent only or 5 µM CT2 (TPE-5) were analyzed by HPLC coupled to light scattering. The cells were loaded with cold OA. Bars represent mean ± s.d., $n$ = 3 wells. $p$-value = 3.57E-6 for TAG and $p$-value = 0.21 for PC estimated by a Student's $t$-test.

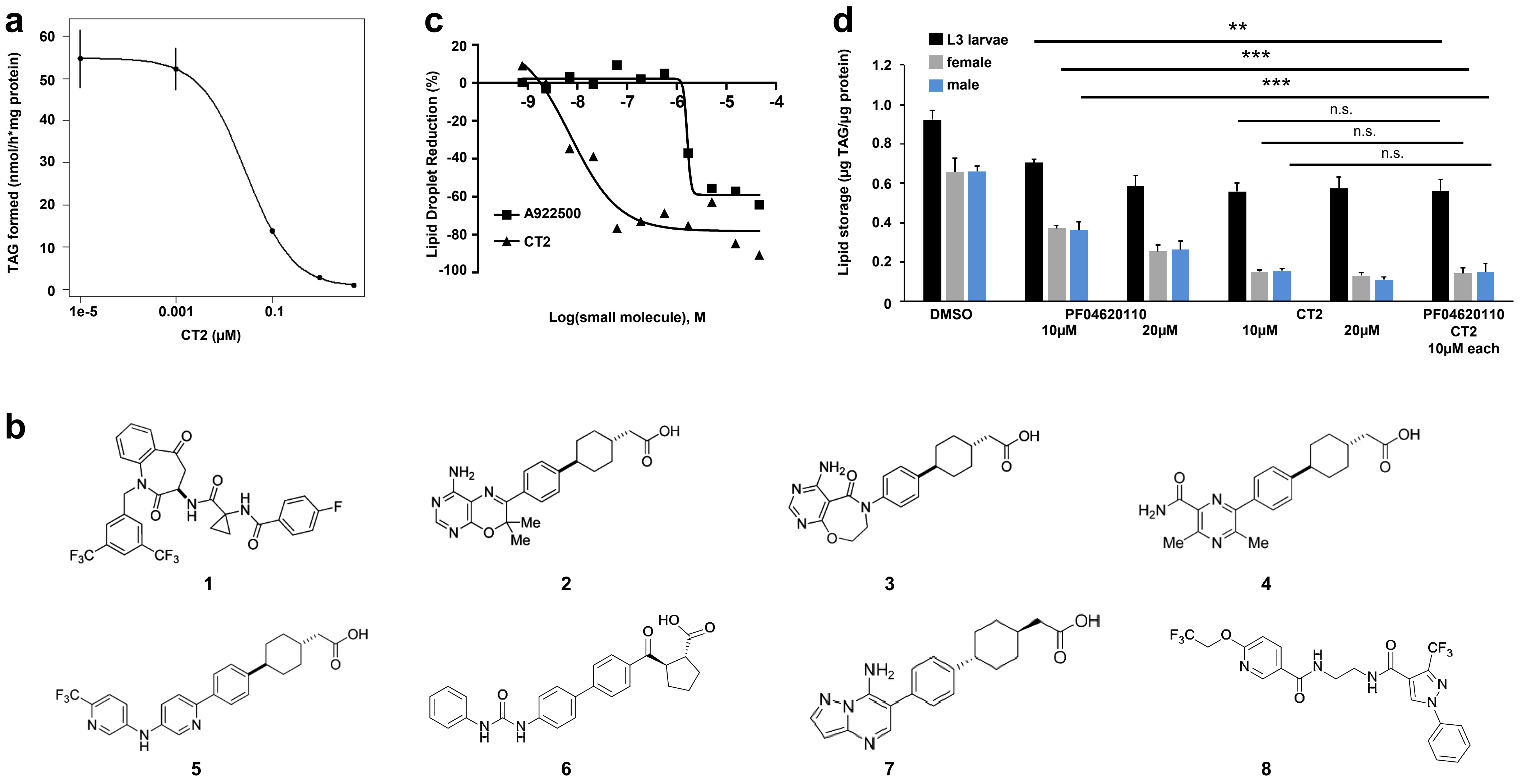
a
TAG formed (nmol/h*mg protein)
60
50
40
30
20
10
0
1e-5
0.001
0.1
CT2 (µM)
c
Lipid Droplet Reduction (%)
20
0
-20
-40
-60
-80
-100
-9
-8
-7
-6
-5
-4
A922500
CT2
Log(small molecule), M
d
L3 larvae
female
male
Lipid storage (µg TAG/µg protein)
1.2
1.0
0.8
0.6
0.4
0.2
0
**
***
***
n.s.
n.s.
n.s.
DMSO
PF04620110
10µM
20µM
CT2
10µM
20µM
PF04620110
CT2
10µM each
b
1
2
3
4
5
6
7
8

# Figure S7

**Fig. S7**, related to Fig. 6: Dose-response curve of CT2 (TPE-5), overview of published DGAT1 small molecule inhibitors, and comparison between CT2 activity and exemplary DGAT1 inhibitors in cells and in vivo. (a) CT2 (TPE-5) inhibits activity of murine DGAT1 expressed in a quadruple yeast mutant which is unable to deposit lipid stores. Experiments were performed as described in Fig. 6G. Data is represented as mean ± s.d., *n* = 3 wells. (b) Published DGAT1 inhibitor structures include: (1) early benzazepinedione compound (Merck) (Liu et al., 2013), (2) T863 (Japan Tobacco/Tularik and Pfizer) (Cao et al., 2011, Fox et al., 2014), (3) PF-04620110 (Pfizer) (Dow et al., 2011), (4) AZD7687 (Astra Zeneca) (McCoull et al., 2012), (5) LCQ908 (Novartis) (Serrano-Wu et al., 2012), (6) A-922500 (Abbott) (Zhao et al., 2008), (7) Compound-14 (Abbott) (Yeh et al., 2012), (8) Compound-A (Takeda pharmaceutical Company Ltd.) (Yamamoto et al., 2011). (c) Dose-response curve for the inhibition of OA-induced lipid storage levels in response to A-922500 or CT2 (TPE-5) in 3T3-L1 cells incubated over night with 400μM OA. Data represent single well measurements. (d) TAG levels of third instar larvae, or sex-separated non-mated 1d old adult flies which were raised on fly food supplemented with PF04620110 or CT2 (TPE-5) alone or in combination (fly experiments were performed as described in Fig. 6c). Bars show mean ± s.d., *n* = 4 times 5 larvae, or 8 flies, respectively. Multiple comparisons were determined by one-way ANOVA followed by Bonferroni's *post-hoc* testing.
