# Supplementary material for: A Class of Diacylglycerol Acyltransferase 1 Inhibitors Identified by a Combination of Phenotypic High-throughput Screening, Genomics, and Genetics
Source: eBioMedicine. 2016 Apr 16;8:49–59. doi: 10.1016/j.ebiom.2016.04.014 (PMC4919474; doi:10.1016/j.ebiom.2016.04.014)
Supplement: Supplementary file 7 — Supplemental Experimental Procedures. [file mmc7.docx]

**Figure S1, related to Figure 1: Quantification of lipid storage amounts in fly cells and the** **activity of CT1 (THI-4), CT2 (TPE-5) and CT3 (AU-6) in Drosophila Kc167 cells.**

Drosophila S3 (a, b) or Kc167 (a,c) cells were incubated with increasing amounts of OA for 16 hours before they were fixed and stained for nuclei/DNA using Hoechst (shown in blue) and LDs by BODIPY493/503 (shown in green). Images were recorded using 20 x magnification and an ImageXpress HCS system (Molecular Devices). Images were analyzed by a custom CellProfiler image segmentation procedure and lipid storage amounts quantified as area of the detected LDs normalized by the area of the DNA/Nuclei. Bars in (b,c) represent mean ± s.d., n = 3 wells. (d) Cells were treated with different concentrations of the respective chemotype as described in Figure 1d. Subsequently, the cells were processed as described in panel (a), and dose-response curves were plotted (e) as well as the number of cells quantified (f). Data in (e,f) represent mean values of three wells per data point. Scale bars represent 50 µm.

**Figure S2, related to Figure 2:** **CT activity is evolutionary conserved.**

Structural derivatives of CTs 1 to 3 were tested in Drosophila S3, monkey COS7 and murine AML12 cells for their ability to block lipid deposition. Structure information can be retrieved from the PubChem database (<https://pubchem.ncbi.nlm.nih.gov/>) using the NCGC IDs (column “CODE”). Cells were treated over night with 5 µM of the respective compound in the presence of 400 µM (S3 and COS7 cells) or 200 µM (AML12 cells) OA, fixed and stained for nuclei and LDs. Images were subsequently recorded, visually inspected and a potential LD phenotype was classified. “N” stands for no change compared to control; “N/A” stands for not analyzed; “reduced” stands for reduced lipid storage levels and “absent” for complete lack of lipid storage levels based on visual inspection of the microscopic images.

**Figure S3, related to Figure 3:** **Quality control of RNA-Seq data.**

(a) A histogram of normalized expression values in the logarithm scale (log (FPKM+1)). The y-axis represents the relative number of genes/intergenic regions within a particular range of expression values (density). The vertical red line represents the 95 percentile of FPKM values for intergenic regions (FPKM 1.04 is used as a cutoff). (b) Replicate correlation matrix including all data. For each sample (e.g. “inactive_CT3”) several replicates were present and used for the clustering. For number of replicates per sample please refer to the materials & methods section. The heatmap color scale is shown on the right and corresponds to Spearman’s correlation coefficient. (c) Replicate correlation matrix including data with Spearman’s coefficient (rho) > 0.95. The heatmap color scale corresponds to Spearman’s correlation coefficient and is recalculated for the data set with Spearman’s coefficient (rho) > 0.95. (d) Inter sample correlation matrix. The heatmap color scale is the same as in panel b.

**Figure S4, related to Figure 3:** **Analysis of differentially expressed (DE) genes.**

(a) Boxplots representing the distribution of DE genes identified in active chemotypes (CT1 = THI-4, CT2 = TPE-69, CT3 = AU-6) vs. DMSO control or inactive chemotypes (CT1 = THI-68, CT2 = TPE-67, CT3 = AU-73) vs. DMSO control. *** - indicates p-value < 0.001 in Wilcoxon signed rank test. The boxplot shows that there are significantly more genes changing their expression in response to active chemotypes than to inactive ones. The number of DE genes for each samples pair is: active_CT1/DMSO_OA – 592, active_CT2/DMSO_OA – 171, active_CT3/DMSO_OA – 284, inactive_CT1/DMSO_OA – 47, inactive_CT2/DMSO_OA – 105, inactive_CT3/DMSO_OA – 49, DMSO_noOA/DMSO_OA – 436. The detailed information is provided in Table S3. (b) Boxplots representing the distribution of DE genes identified in active chemotypes vs. DMSO control, inactive chemotypes vs. DMSO control, and DE genes that are common for both active and inactive vs. DMSO control along with their direction of change (yellow = increased expression, blue = decreased expression). These boxplots show that the number of DE genes changing in active chemotypes vs. DMSO control is higher than in the other two conditions indicating specificity of DE gene response in active chemotypes. (c) A heatmap of Modulated Modularity Clustering of 15 different functional modules of DE genes identified in the comparison “active chemotypes vs. DMSO”. Black numbers in the heatmap indicate modules. Color code corresponds to Pearson’s (r) correlation coefficient, with the heatmap scale shown on the right. Gene lists from each module are presented in Table S4. (d) GO terms enriched for genes belonging to the clusters 1, 3, 4 or 5 shown on the heatmap in Figure 3a. There was no significant enrichment of GO-terms for cluster 2. For details see Table S5. (e) Gene Ontology (GO) terms enriched for DE genes identified in each active chemotype (CT1, CT2, and CT3) vs. DMSO. Analysis was done using the GOrilla software based on the exact minimum hypergeometric (mHG) p-value algorithm and the REViGO program to filter secondary GO terms.

**Figure S5, related to Figure 5: CT2 (TPE-5) blocks lipid deposition during the differentiation of human stem cells and in differentiated human myotubes.**

(a) hMSCs deposit large amounts of lipids during differentiation. If CT2 (TPE-5) is added pre-induction, or at the different induction rounds, this lipid deposition is efficiently blocked. Green: LDs (BODIPY493/503), blue: DNA/nuclei (DAPI). Scale bar represents 30 µm. (b) CT2 (TPE-5) blocks lipid deposition in differentiating hMSCs derived of the Mid-Atlantic Nutrition Obesity Research Center (NORC). hMSCs deposit large amounts of lipids during the differentiation as revealed by bright field images or OilRedO staining quantification (c). Treatment of the cells with 1 µM CT2 (TPE-5) during the differentiation process resulted in a block of lipid deposition. Images were recorded with a standard tissue culture microscope with a 10x objective. Bars represent mean ± SEM, n = 2 wells. Pairwise comparison statistics was determined by Student’s t-test (p-value = 0.0014). (d) Differentiated myotubes obtained from a muscle biopsy of a male type 2 diabetes patient deposit LDs when they are provided with 100 µM OA in the presence of DMSO only, or 5 µM of an inactive CT2 structure (TPE-67). When 5 µM of the active CT2 structure TPE-5 were present, however, lipid deposition was prominently blocked. Cells were stained with DAPI for DNA/nuclei and BODIPY493/503 for LDs. Scale bar represents 1 µm.

**Figure S6, related to Figure 5:** **CT2 does not block cholesterylester droplet deposition in AML12 cells and causes a TAG-specific lipid storage phenotype in COS-7 cells.**

(a) AML12 cells were incubated with NBD-cholesterol (shown in green) in the absence or presence of OA and DMSO or 5 µM CT2 (TPE-5) for 18 hours. Subsequently, the cells were fixed, stained for DNA/nuclei (Hoechst33342 shown in blue) and LDs (LipidTOX HCS Deep Red, shown in red) and analyzed by microscopy. Scale bar represents 50 µm. (b) Thin layer chromatography of COS7 cell lipid extracts. The cells were treated either with DMSO only or with 5 µM CT2 (TPE-5) and lipid deposition was induced by providing radiolabeled OA. (c) Lipid extracts of COS7 cells treated with the DMSO solvent only or 5 µM CT2 (TPE-5) were analyzed by HPLC coupled to light scattering. The cells were loaded with cold OA. Bars represent mean ± s.d., n = 3 wells. p-value = 3.57E-6 for TAG and p-value = 0.21 for PC estimated by a Student’s t-test.

**Figure S7, related to Figure 6:** **Dose-response curve of CT2 (TPE-5), overview of published DGAT1 small molecule inhibitors, and comparison between CT2 activity and exemplary DGAT1 inhibitors in cells and in vivo.**

(a) CT2 (TPE-5) inhibits activity of murine DGAT1 expressed in a quadruple yeast mutant which is unable to deposit lipid stores. Experiments were performed as described in Figure 6G. Data is represented as mean ± s.d., n = 3 wells. (b) Published DGAT1 inhibitor structures include: (1) early benzazepinedione compound (Merck) (Liu et al., 2013), (2) T863 (Japan Tobacco/Tularik and Pfizer) (Cao et al., 2011, Fox et al., 2014), (3) PF-04620110 (Pfizer) (Dow et al., 2011), (4) AZD7687 (Astra Zeneca) (McCoull et al., 2012), (5) LCQ908 (Novartis) (Serrano-Wu et al., 2012), (6) A-922500 (Abbott) (Zhao et al., 2008), (7) Compound-14 (Abbott) (Yeh et al., 2012), (8) Compound-A (Takeda pharmaceutical Company Ltd.) (Yamamoto et al., 2011). (c) Dose-response curve for the inhibition of OA-induced lipid storage levels in response to A-922500 or CT2 (TPE-5) in 3T3-L1 cells incubated over night with 400µM OA. Data represent single well measurements. (d) TAG levels of third instar larvae, or sex-separated non-mated 1d old adult flies which were raised on fly food supplemented with PF04620110 or CT2 (TPE-5) alone or in combination (fly experiments were performed as described in Fig. 6c). Bars show mean ± s.d., n = 4 times 5 larvae, or 8 flies, respectively. Multiple comparisons were determined by one-way ANOVA followed by Bonferroni’s *post-hoc* testing.

**Supplemental Tables**

Table S1: RNA-Seq gene expression values (FPKM values) for each separate sample.

Table S2: RNA-Seq gene expression values (FPKM values) per sample.

Table S3: CuffDiff results indicating significance of differential gene expression between two samples.

Table S4: The table summarizes the results for 919 differentially expressed genes used for the clusters shown in Figure 3a and Figure S4c

Table S5: Details of the GO-class enrichment of the clusters shown in Figure 3a. Table relates also to Figure S4d.

**Supplemental Experimental Procedures**

qHTS screening:

The primary qHTS was performed with embryonic Drosophila S3 cells (Bloomington Drosophila Stock Center [DGRC]), that showed the highest dynamic range between the unfed and OA-induced state and best performance (such as viability and adherence) during automated liquid handling in 1,536-well format. We dispensed 4 µL of cells at 1.25 x 10^6^ cells/ml into LoBase Aurora COC 1,536-well plates (black walled, clear bottom) with a bottle-valve solenoid-based dispenser (Aurora, Carlsbad, CA, USA) to obtain 5,000 cells/well. A total of 23 nL of compound solution of different concentrations were transferred to the assay plates using a Kalypsys pin tool equipped with a 1,536-pin array containing 10-nL slotted pins (FP1S10, 0.457-mm diameter, 50.8 mm long; V&P Scientific, San Diego, CA, USA). One microliter of OA (400 µM) was added, and the plate was lidded with stainless steel rubber gasket-lined lids containing pinholes. The final concentration of the library compounds in the 5 µL assay volume ranged from 46 μM to 3 nM in 1:5 dilution steps. The positive control of lipid storage inhibition used in the primary screen is the known long chain fatty acyl-CoA synthetase inhibitor Triacsin C (Igal et al., 1997). After 18–24 h incubation at 24 °C and 95% humidity, BODIPY 493/503 (Molecular Probes – Thermo Fisher Scientific Inc., Waltham, MA, USA) was added to the wells to stain lipid droplets, and the Cell Tracker Red CMTPX dye (Molecular Probes – Thermo Fisher Scientific Inc., Waltham, MA, USA) was added to enumerate cell number. Fluorescence was detected by excitation of the fluorophores with a 488 nm laser on an Acumen Explorer (TTP Lab Tech, Hertfordshire, UK). The total intensity in channel 1 (500–530 nm) reflected lipid droplet accumulation. Cells were detected using channel 3 (575–640 nm) with 5-lm width and 100-lm depth filters. The ratio of the total intensity in PMT channel 1 over total intensity of channel 3 was also calculated. Percent activity was calculated relative to DMSO basal (0%) and the positive control (100% inhibited lipid droplet deposition due to the presence of 20 µM Triacsin C). All screening data is available at the PubChem repository with assay ID 2685.

Data analysis and clustering of compounds by chemical structures:

To determine compound activity in the qHTS assay, the concentration-response data for each sample was plotted and modeled by a four parameter logistic fit yielding IC50 and efficacy (maximal response) values as previously described (Inglese et al., 2006). Data normalization and curve fitting were performed using in-house informatics tools. Compounds were designated as Class 1–4 according to the type of concentration–response curve (CRC) observed. Usually the qHTS screen yielded hits with a wide range of potencies and with substantial variation in the quality of the corresponding CRCs (efficacy and number of asymptotes), which included samples associated with shallow curves or single-point extrapolated concentration responses; these were assigned as low-confidence activities. In brief, Class 1.1 and 1.2 were the highest-confidence complete CRCs containing upper and lower asymptotes with efficacies ≥ 80% and < 80%, respectively. Class 2.1 and 2.2 were incomplete CRCs having only one asymptote with efficacy ≥ 80% and < 80%, respectively. Class 3 CRCs showed activity at only the highest concentration or were poorly fit. Class 4 CRCs were inactive having a curve-fit of insufficient efficacy or lacking a fit altogether.

Compounds from the primary qHTS screen were further classified into three categories according to the quality of curve fit and efficacy. Compounds that showed activation/inhibition in both the ratio and the 500–530 nm readouts were defined as activators/inhibitors. High-quality actives: compounds in curve class 1.1, 1.2, 2.1 and 2.2 curves with efficacy higher than 60%; inactives: compounds with class 4 curves; inconclusive: all other compounds including those shallow curves and curves with single point extrapolated activity.

High-quality active compounds were clustered based on structural similarity to identify common chemotypes using the Leadscope software. Hierarchical agglomerative clustering method with complete linkage and a distance of 0.7 Tanimoto cutoff was performed in the analysis.

Chemical syntheses:

**General Methods for Chemistry.** All air or moisture sensitive reactions were performed under positive pressure of nitrogen with oven-dried glassware. Anhydrous solvents such as dichloromethane, *N,N*-dimethylforamide (DMF), acetonitrile, dioxane, dimethoxyethane, methanol and triethylamine were purchased from Sigma-Aldrich (St. Louis, MO, USA). Preparative purification was performed on a Waters semi-preparative HPLC system using a Phenomenex Luna C18 column (5 micron, 30 x 75 mm) at a flow rate of 45 mL/min. The mobile phase consisted of acetonitrile and water (each containing 0.1% trifluoroacetic acid). A gradient of 10% to 50% acetonitrile over 8 minutes was used during the purification. Fraction collection was triggered by UV detection (220 nm). Analytical analysis was performed on an Agilent LC/MS (Agilent Technologies, Santa Clara, CA, USA). Final QC LCMS Method: A 7 minute gradient of 4% to 100% Acetonitrile (containing 0.025% trifluoroacetic acid) in water (containing 0.05% trifluoroacetic acid) was used with an 8 minute run time at a flow rate of 1 mL/min. A Phenomenex Luna C18 column (3 micron, 3 x 75 mm) was used at a temperature of 50° C. Purity determination was performed using an Agilent Diode Array Detector for both Method 1, Method 2 and Method 3. Mass determination was performed using an Agilent 6130 mass spectrometer with electrospray ionization in the positive mode. ^1^H NMR spectra were recorded on Varian 400 MHz spectrometers. Chemical shifts are reported in ppm with undeuterated solvent (DMSO-*d*6 at 2.49 ppm) as internal standard for DMSO-*d*6 solutions. All of the analogs tested in the biological assays have purity greater than 95%, based on both analytical methods**.** High resolution mass spectrometry was recorded on Agilent 6210 Time-of-Flight LC/MS system. Confirmation of molecular formula was accomplished using electrospray ionization in the positive mode with the Agilent Masshunter software (version B.02)

**Synthesis of compound 4**

A solution of cyclohexane-1,3-dione (5 g, 44.6 mmol) and of 1,1-dimethoxy-*N,N*-dimethylmethanamine (20 mL, 150 mmol) was stirred under reflux for 2h. After evaporation, the crude product was purified by Biotage (0-5% MeOH-DCM) to obtain desired product as solid (4.0 g, 53.6%).

To the solution of 2-((dimethylamino)methylene)cyclohexane-1,3-dione (2 g, 11.96 mmol) in MeOH (70 ml) and Water (70.0 ml) was added (4-methoxyphenyl)hydrazine hydrochloride (2.089 g, 11.96 mmol), followed by NaOH (0.478 g, 11.96 mmol). The mixture was reflux for 2hrs, removed solvent, added AcOH (6.85 ml, 120 mmol) and then Water (70.0 ml). The mixture was heated at 110oC for 2 hrs. The solvent was removed. The residue was dissolved in EtOAc and washed with Sat. NaHCO3 (3X) and brine. The organic layer was dried over Na2SO4 and concentrated. The crude product was purified by Biotage (10%-60% EtOAc/hexane) to give desired product as solid (2.0 g, 69.0%).

To the suspension of 1-(4-methoxyphenyl)-6,7-dihydro-1H-indazol-4(5H)-one (2 g, 8.26 mmol) in *i*PrOH (80 ml) was added ammonium acetate (6.36 g, 83 mmol). The mixture was stirred at r.t. for 2hrs. To the reaction mixture were added 4A mol sieves (powder) (2g) and sodium cyanoborohydride (2.59 g, 41.3 mmol). The mixture was stirred at 70 °C for 6hrs. The mixture was filtered and the solvent was removed under reduced pressure. The residue was diluted with EtOAc and washed with 1N NaOH and brine, dried over Na2SO4, and concentrated. The crude product was purified by Biotage (9/1/0.1 DCM/MeOH/TEA) to obtained desired product (1.5g, 75%).

To a solution of picolinic acid (0.308 g, 2.503 mmol) in DMF (25 ml) was added HATU (1.047 g, 2.75 mmol) and diisopropylethylamine (1.312 ml, 7.51 mmol). The solution was stirred at r.t. for 10 min, and then 1-(4-methoxyphenyl)-4,5,6,7-tetrahydro-1H-indazol-4-amine (0.67 g, 2.75 mmol) was added. The reaction mixture was stirred at r.t. for overnight. Water (100 ml) was added to the mixture. The solid was filtered and washed with water, and dried. The crude product was purified by Biotage (20-60% EtOAc/hexanes) gave the title compound (0.69g, 79%).

To a solution of N-(1-(4-methoxyphenyl)-4,5,6,7-tetrahydro-1H-indazol-4-yl)picolinamide (0.09 g, 0.258 mmol) in DCM (3 ml) was dropwise added BBr_3_ (0.775 ml, 0.775 mmol) (1M in DCM) at 0oC. The reaction mixture was stirred at r.t. for overnight. After the reaction was completed, water (10ml) was added to the reaction mixture at 0oC. The mixture was extracted with EtOAc (3x10ml). The combined organic layers was washed with brine, dried over Na2SO4, and concentrated. The crude product was purified by Biotage (30-100% EtOAc/ hexanes) gave the title compound (29 mg, 33.6%).

**N-(1-(4-hydroxyphenyl)-4,5,6,7-tetrahydro-1H-indazol-4-yl)picolinamide**

^1^H NMR (400 MHz, DMSO-*d*_6_) δ ppm 8.62 - 8.69 (m, 2 H), 8.12 - 8.18 (m, 1 H), 8.09 (t, *J*=7.63 Hz, 1 H), 7.66 (dd, *J*=7.04, 5.48 Hz, 1 H), 7.47 - 7.52 (m, 1 H), 7.28 - 7.36 (m, 2 H), 6.80 - 6.94 (m, 3 H), 5.10 - 5.21 (m, 1 H), 2.57 - 2.77 (m, 2 H), 1.68 - 2.05 (m, 4 H); Method 1, retention time: 4.493 min; HRMS: *m/z* (M+H)^+^ = 335.1501 (Calculated for C_19_H_19_N_4_O_2_ = 335.1503).

**Synthesis of compound 5**

To a solution of 4H-thieno[3,2-b]pyrrole-5-carboxylic acid (0.2 g, 1.196 mmol) in DMF (10 ml) was added HATU (0.5 g, 1.316 mmol) and DIPEA (0.836 ml, 4.79 mmol). The mixture was stirred at r.t. for 30 min, and then 2-(3,4-diethoxyphenyl)ethanamine (0.275 g, 1.316 mmol) was added. The reaction was stirred at r.t. for overnight. Water was added to the mixture, and extracted with EtOAc. The organic layer was dried over MgSO4 and concentrated. The crude product was purified by Biotage (0-10%, MeOH /DCM) to give the title product (0.25 g, 58.3%).

To the solution of N-(3,4-diethoxyphenethyl)-4H-thieno[3,2-b]pyrrole-5-carboxamide (30 mg, 0.084 mmol) in DMF (1.5 ml) was added sodium hydride (60%)(3.68 mg, 0.092 mmol). The mixture was stirred at r.t. for 1h. 4-(Bromomethyl)-1-chloro-2-fluorobenzene (22.44 mg, 0.100 mmol) was added to the mixture. The reaction mixture was stirred at r.t. for overnight. The crude product was purified by reverse phase purification system to give the title product as a TFA salt (10.5 mg, 20.4%).

**4-(4-Chloro-3-fluorobenzyl)-N-(3,4-diethoxyphenethyl)-4H-thieno[3,2-b]pyrrole-5-carboxamide**

^1^H NMR (400 MHz, DMSO-*d*_6_) δ ppm 8.35 (t, *J*=5.67 Hz, 1 H), 7.49 (t, *J*=8.02 Hz, 1 H), 7.45 (d, *J*=5.48 Hz, 1 H), 7.15 - 7.22 (m, 2 H), 7.13 (s, 1 H), 6.93 (dd, *J*=8.22, 1.57 Hz, 1 H), 6.82 (d, *J*=7.83 Hz, 1 H), 6.79 (d, *J*=1.96 Hz, 1 H), 6.66 (dd, *J*=8.22, 1.96 Hz, 1 H), 5.77 (s, 2 H), 3.95 (qd, *J*=7.04, 5.09 Hz, 4 H), 3.37 - 3.44 (m, 2 H), 2.72 (t, *J*=7.24 Hz, 2 H), 1.27 (dt, *J*=12.42, 6.90 Hz, 6 H); Method 1, retention time: 6.951 min; HRMS: *m/z* (M+H)^+^ = 501.1389 (Calculated for C_26_H_27_ClFN_2_O_3_S = 501.1409).

**Synthesis of compound 6**

To a solution of 2-(tert-butoxycarbonylamino)-2-methylpropanoic acid (2.187 g, 10.76 mmol) in DMF (35 ml) was added HATU (4.5 g, 11.84 mmol) and diisopropylethylamine (5.64 ml, 32.3 mmol). The mixture was stirred at r.t. for 30 min, and then ethyl piperidine-4-carboxylate (1.823 ml, 11.84 mmol) was added. The reaction was stirred at r.t. for 6 hrs. Water was added to the mixture, and extracted with EtOAc. The organic layer was dried over MgSO4 and concentrated. The crude product was used in the next reaction without further purification (3.6g, 98%).

To a solution of ethyl 1-(2-(tert-butoxycarbonylamino)-2-methylpropanoyl)piperidine-4-carboxylate (3.6 g, 10.51 mmol) in DCM (40 ml) was added TFA (8 ml, 104 mmol). The mixture was stirred at r.t. for 4 hrs. The solvent was evaporated. The crude product was used in the next reaction without further purification (1.8g, 71%).

To the solution of ethyl 1-(2-amino-2-methylpropanoyl)piperidine-4-carboxylate (50 mg, 0.206 mmol) and diisopropylethylamine (0.072 ml, 0.413 mmol) in DMF (1.5 ml) was added 2,4-dichloro-1-isocyanatobenzene (38.8 mg, 0.206 mmol). The mixture was stirred at r.t. for 1hr. The reaction was completed. The crude product was purified by reverse phase purification system to give the title product as a TFA salt (59.9 mg, 53.3%).

**Ethyl 1-(2-(3-(2,4-dichlorophenyl)ureido)-2-methylpropanoyl)piperidine-4-carboxylate**

^1^H NMR (400 MHz, DMSO-*d*_6_) δ ppm 8.11 (d, *J*=9.00 Hz, 1 H), 8.07 (s, 1 H), 7.55 (d, *J*=2.35 Hz, 1 H), 7.50 (s, 1 H), 7.30 (dd, *J*=9.00, 2.74 Hz, 1 H), 4.29 (d, *J*=13.30 Hz, 2 H), 3.99 (q, *J*=7.04 Hz, 2 H), 2.84 - 2.97 (m, 2 H), 2.51 - 2.57 (m, 1 H), 1.76 (dd, *J*=13.30, 3.13 Hz, 2 H), 1.32 - 1.45 (m, 8 H), 1.09 (t, *J*=7.04 Hz, 3 H); Method 1, retention time: 5.494 min; HRMS: *m/z* (M+H)^+^ = 430.1287 (Calculated for C_19_H_26_Cl_2_N_3_O_4_ = 430.1295).

Drosophila handling and compound treatment:

*Drosophila melanogaster* flies mutant for the white gene (*white[*]*) were used as reference fly strain. Additionally, homo-or heterozygous mutants of the *brummer[1]* (Grönke et al., 2005), *Lsd-2/PLIN2[51]* (Grönke et al., 2003) and *midway/dmDGAT1[QX25]* (Buszczak et al., 2002) genes were used. Flies were kept at 25 °C with a 12hours day / night cycle unless otherwise noted on a standard cornmeal / molasses diet.

For compound treatments, flies were kept / developed on freshly prepared fly food containing either DMSO only or 10 µM of the indicated compound solved in DMSO. The fly food used consisted of (per 100 mL total volume): 0.624 g Agar, 8 g polenta, 1 g soy flour, 1.8 g dry yeast, 2.2 g treacle, 8 g malt extract and 1.5 mL nipagin solution (10% in Ethanol) and 0.63 mL propionic acid. After preparation, the complete food was cooled down to approximately 60°C in a water bath before either DMSO alone or compound dissolved in DMSO was added (final concentration of DMSO was 0.2 %). Subsequently, the medium was shaken rigorously to mix the compound with the food and poured in fly vials where it solidified. Flies were placed on the food for two to three days and subsequently removed. After the indicated incubation time, the progeny were collected and triglyceride measurements were performed according to published protocols (Hildebrandt et al., 2011). In brief, we collected in at least three biologically independent experiments three times 5 larvae or 8 adult male flies per genotype/treatment and homogenized them in water including 0.05% Tween-20. The extracts were heat inactivated and aliquots were incubated with triglyceride assay reagent (Thermo Fisher Scientific, Passau, Germany) to measure the glycerol backbone of the neutral storage lipids which are mainly consisting of TAG or a standard BCA protein determination assay (Thermo Fisher Scientific Inc., Waltham, MA, USA ) for normalization purposes. Colorimetric measurements were quantified using a BioTek Synergy Mx multimode plate reader. In order to test for a compound effect in diet-induced obesity we included 10% coconut oil (Sigma-Aldrich, St. Louis, MO, USA) in the food (Shirazi et al., 2014). Flies kept on coconut oil containing food were incubated at 18 °C since at higher temperatures flies became trapped in the liquefied coconut oil.

Tissue culture handling:

*Drosophila cells*

Drosophila Kc167 cells were obtained from the Harvard RNAi screening center and Drosophila S3 cells were obtained from the Drosophila Genomics Resource Center (DGRC). Drosophila cells were cultured in Schneider’s medium (PAN Biotech, Aidenbach, Germany or Gibco/BRL by Thermo Fisher Scientific Inc., Waltham, MA, USA) including 10% FCS (PAN Biotech) and supplemented with Penicillin/Streptomycin (Gibco/BRL) at 25°C and without CO2. For compound experiments, the cells were treated in serum reduced medium (5% FCS instead of 10%) in 6-well plates (Sarstedt, Nuembrecht, Germany), 8-well chamber slides (Sarstedt) or 96-well plates (Greiner BioOne, Frickenhausen, Germany). Oleic acid (Calbiochem, Schwalbach, Germany) bound to fatty acid free BSA (Sigma-Aldrich) solved in Tris pH 8.0 was added to the cells at the given concentrations to induce lipid storage deposits.

*Mouse AML12 hepatocytes*

AML12 cells were obtained from ATCC (Manassas, VA; CRL-2254) and grown according to the standard protocol (Hauner et al., 2001).

*Differentiation of mouse 3T3-L1 fibroblasts to adipocytes*

3T3-L1 cells (ATCC, CL-173) were seeded in 96-well optical quality cellBind assay plates with 300 cells / well in 200µL. The cells were kept in preadipocyte medium (Zen-Bio, Research Triangle Park, NC, USA) until they reached 100% confluency. Afterwards, the medium was replaced with differentiation medium (Zen-Bio, Research Triangle Park, NC, USA; the medium contains biotin, panthothenate, human insulin, dexamethasone, isobutylmethylxanthine and a PPARγ agonist) and the cells were incubated for three days before the differentiation medium was replaced by post-differentiation medium. After four more days, cells were fixed and stained as described below.

*African green monkey COS7 kidney cells*

SV-40 transformed monkey embryonic kidney cells (COS7; ATCC, CRL-1651) were cultivated in Dulbecco´s modified eagle medium (DMEM; GIBCO/BRL), containing 10% fetal calf serum (FCS), penicillin (100 IU/ml, GIBCO/BRL), and streptomycin (100 μg/ml, GIBCO/BRL) with 95% humidified atmosphere, 37°C and 7.5% CO2.

*Differentiation of human visceral pre-adipocytes*

Human visceral pre-adipocytes (LONZA, Basel, Switzerland) were seeded in 96-well plates (10,000 cells per well in 200µL) with pre-induction medium (LONZA, PBM-2). After two days we began three rounds of differentiation induction for two days each by adding the PGM-2 (LONZA) medium. Finally, cells were kept in PGM-2 (LONZA) medium for five more days before they were fixed and stained as described below.

*Differentiation of human mesenchymal stem cells (2 sources)*

- Cells obtained from LONZA

Human mesenchymal stem cells were seeded in 96-well plates (7,000 cells /well in 100µL). After an overnight accommodation phase the standard medium was removed and maintenance medium including DMSO or 800nM TPE-5 was added for three days (pre-induction treatment). Afterwards, the maintenance medium was replaced with induction medium including either DMSO or 800nM of the TPE-5 compound (induction round 1 treatment paradigm). After three days, the induction medium was removed and the cells were allowed to recover for three days in fresh maintenance medium. This induction / recovery cycle was subsequently repeated two times. At the end, the cells were fixed and stained as described below.

- Cells obtained from NORC

Human mesenchymal stem cells (hMSCs) isolated from adipose stromal cell fraction were obtained from the Mid Atlantic NORC biobank and seeded in 35 mm dishes, grown in α-minimum essential medium (MEM) with 10% FBS, 100 units/ml penicillin, and 100 μg/ml streptomycin. Fully confluent preadipocytes (day 0) were then differentiated for 13 days using a serum-free complete differentiation medium (Dulbecco’s modified Eagle’s medium (DMEM)/F12 with 0.5 mmol/l IBMX, 100 nmol/l insulin, 100 nmol/l dexamethasone, 2 nmol/l T3, 10 μg/ml transferrin, 1 μmol/l ROSI, 33 μmol/l biotin, and 17 μmol/l pantothenic acid). After 3 days, cells were maintained in DMEM/F12 medium supplemented with insulin (10 nmol/l) and dexamethasone (10 nmol/l). All media were supplemented from day 0 on with DMSO or CT2/TPE-5. Cells were differentiated without a source of exogenous lipids. Differentiation efficiency is measured by Oil Red O (Sigma-Aldrich) staining, extraction and quantification at Day 13 post-differentiation. Study protocols to obtain hMSCs were approved by the Institutional Review Board at the University of Maryland, and all subjects gave written informed consent.

*Differentiation of human myotubes from a type 2 diabetes subject muscle biopsy*

Satellite cells were established from muscle biopsy samples of a vastus lateralis biopsy of a male type 2 diabetes patient. For this purpose, muscle cell cultures free of fibroblasts were established by the method of Henry (Henry et al., 1995). Briefly, muscle tissue was dissected in Ham’s F-10 medium at 4°C and dissociated by three successive treatments with 0.05% trypsin/EDTA. Satellite cells were resuspended in skeletal muscle growth medium (SKGM Cat cc-3161 from Lonza) supplemented with 10% FBS and SKGM SingleQuots (Cat# CC-4139 from LONZA) which contains rhEGF, fetuin, DEX, BBSA, Gentamicin, Amphotericin and insulin as described by the manufacturer. The cells were grown on culture wells or flasks coated with extracellular matrix gel with growth medium as above minus insulin and DEX. After 1–2 weeks and at ~80% confluence, the growth medium was replaced by DMEM phenol red free medium including 2% horse serum and 30 ug/ml Gentamicin and 15 ng/ml Amphotericin to induce the differentiation of myoblasts into multinucleated myotubes. The cells were cultured in a humidified 5% CO2 atmosphere at 37°C, and medium was changed every two to three days. All myotube cultures were used for analysis on day 8 or 9 after the onset of differentiation. For this purpose, they were incubated with 100µM OA and DMSO only or 5µM of active or inactive CT2 derivatives overnight. Afterwards, the cells were fixed and stained as described below. The Institutional Review Board of the University of Maryland approved all methods and procedures. Each participant provided written informed consent to participate in the study.

The cell lines were treated with different amounts of OA in order to safeguard the best signal to background ratio as well as to acknowledge their different metabolic characteristics. We used the following maximum OA amounts and timespans without noting adverse effects of the OA addition to the cells:

- Drosophila S3 cells: up to 2 mM OA for 18 hours
- Drosophila Kc167 cells: up to 2 mM OA for 18 hours
- Murine AML12 cells: up to 200 µM OA for 18 hours
- African green monkey COS7 cells: up to 400 µM OA for 18 hours
- Human HeLa cells: up to 400 µM OA for 18 hours
- Human primary differentiated muscle cells: up to 200 µM OA for 18 hours

Fluorescent staining of neutral lipids (LDs) and DNA (nuclei) in cells:

At the end of the respective experiment, cells were washed with PBS, fixed with 4 or 5% paraformaldehyde, washed again with PBS and stained with Hoechst33258, Hoechst33342, or DAPI for the DNA and BODIPY493/503 for the neutral lipids (all dyes from Molecular Probes). In some experiments, the cytoplasmic stain HCS CellMask deep red (Molecular Probes) was additionally used. Images were acquired with different imaging systems: ImageXpress high content imaging system (Molecular Devices, Sunnyvale, CA, USA) with a 20 x magnification; an InCell-2000 automated microscope (GE Healthcare, Solingen, Germany) using a 20 x objective or a confocal microscope and a 40 x water-, or 63 x oil-immersion, objective.

For the lipid storage quantifications shown in Figure S1 images were processed with a custom CellProfiler script (available upon request). The amount of detected LDs (particle number or area) was normalized by the cell number (nuclei number or area) and used as a proxy for the cellular lipid storage amounts. Dose-response curves were analyzed using R ([www.r-project.org](http://www.r-project.org)) and the drc package (Ritz and Streibig, 2005).

Electron microscopy:

Drosophila Kc167 cells were grown in 6-well plates with the indicated medium supplements (only DMSO, DMSO and 800 µM OA, 800 µM OA and 5 µM TPE-5). After 18 hours incubation time, the cells were harvested, washed with PBS and 0.1 M Na2HPO4/NaH2PO4 pH7.2 (Sörensen PO4 buffer) before they were fixed with 2.5% glutaraldehyde in 0.1 M PO4 buffer. After another wash with PO4 buffer, the cells were treated with 2% osmium before they were washed again with PO4 buffer and dH2O and afterwards treated with 2% uranylacetate. In the following, the cells were washed again with dH2O and dehydrated by an ethanol, acetone, acetone:araldite (1:1) series before the acetone was evaporated and the cells were mounted in araldite. Ultrathin sections were done with a Reichert OM U3 microtome and images were acquired with a Zeiss EM 109EM and a CCD/Tröndle camera with the settings Image SP.

NBD-cholesterol feeding:

For insect cells: Drosophila S3 cells were seeded in 8-well chamber slides (Sarstedt) in serum reduced Schneider’s medium (5% FCS) and 5 µM of the respective compounds or DMSO only as a control. One well was treated with DMSO and 800 µM OA. After two hours the cells were serum starved for 45 min before 1 µg/mL NBD-cholesterol (Molecular Probes) in serum reduced Schneider’s medium was added. Three hours later the cells were imaged by confocal microscopy using a 40 x water immersion objective and 488 nm excitation and an emission detection from 493-589 nm. Z-stacks were recorded and used for maximum intensity projections.

For mammalian AML12 cells: AML12 cells were plated in optical quality 96-well plates and incubated for one day. Then 100 µL of serum reduced medium (5% FCS) including the small molecules (5 µM in DMSO) and 1 µg/mL NBD-cholesterol was added either with or without 400 µM OA. After 18 hours treatment, the cells were fixed and stained with BODIPY493/503 for LDs and Hoechst33342 for DNA/nuclei (both dyes from Molecular Probes).

Radiolabeled fatty acid uptake experiment:

Confluent COS7 cells (in 6-well plates) were incubated for different time periods with cultivation media, containing 200 µM oleic acid, 0.5 µCi ^3^H-labeled oleic acid/ml, and 5 µM TPE-5 (dissolved in DMSO) or DMSO alone. Subsequently, cells were washed three times with phosphate-buffered saline (PBS) and lysed by incubation with NaOH/SDS (0.3N/0.1%) for 3 h under steady shaking on RT. Radioactivity contained in cell lysates were determined by scintillation counting.

Qubit fluorescent fatty acid uptake assay:

Drosophila S3 cells were seeded in 8-well chamber slides (Sarstedt) in serum reduced Schneider’s medium (5% FCS) and 5 µM of the respective compounds or DMSO only as a control. One well was treated with DMSO and 800 µM OA. After two hours the cells were serum starved for 45 min before a QBT (Molecular Devices) working dilution in Hanks’ Balanced Salt Solution (HBSS) was added according to the manufacture’s descriptions. Two hours later cells were imaged by confocal microscopy using a 40 x water immersion objective and 488 nm excitation and an emission detection from 493-589 nm. Z-stacks were recorded and used for maximum intensity projections.

Lipid extraction and thin layer chromatography:

Drosophila Kc167 cells were grown in the presence or absence of 5 µM of the respective compound in DMSO or DMSO only in 6-well plates. After 18 hours incubation cells were washed with PBS, resuspended in PBS and lipids were extracted by chloroform/methanol (Dole extraction). Lipids were separated on silica plates (Merck, Darmstadt, Germany) using either chloroforme:acetone:acetic acid (45:4:0.5) for neutral lipids or chloroforme:methanol:acetic acid:dH_2_O (25:15:4:2) for phospholipids, impregnated with CuSO4 in phosphoric acid and visualized by charring at 120°C.

For the pre-feeding experiment shown in Figure 5F, Drosophila Kc167 cells were seeded in 6-well plates and incubated over night with 400 µM OA before the OA medium was replaced with serum free medium including 5 µM of the respective compound. After four more hours, the cellular lipids were extracted and separated as described above.

Cell Loading, total lipid extraction and separation (TLC/HPLC-ELSD)

Confluent COS7 cells (in 10 cm plates) were incubated with cultivation media containing 200 µM OA (in some cases 0.6 µCi ^14^C-labeled OA was added as tracer) in the presence or absence of 5 µM TPE-5 for 12 h. Subsequently, cells were washed three times with PBS and lipids were extracted twice with hexane/2-propanol (1/1, v/v). Organic phases were combined and evaporated. Lipid extracts of cells incubated with ^14^C-labeled OA were dissolved in 100 µl chloroform and separated by TLC using chloroform/acetone/acetic acid (90/8/1; v/v/v; neutral lipids) or chloroform/methanol/acetone/acetic acid/water (50/10/20/15/5; v/v/v/v/v; phospholipids) as solvents. Radiolabeled lipids were detected using a storage phosphor screen and the chemiluminescence signal analyzed using a molecular imager system (Storm 860, GE). Extracts of “non-^14^C-labeled” cells were dissolved in 400 µl chloroform/methanol (1/1, v/v) and analyzed by HPLC-ELSD. For normalization cells were lysed in NaOH/SDS (0.3N/0.1%) for 3 h under steady shaking on RT and protein amounts were quantified by standard assays.

Determination of DGAT Activity

Homogenates (50 µg protein) of YJP1078 *S. cerevisiae* quadruple knock-out cells (MATαhis3∆1 leu2∆0 lys2∆0 ura3∆0 ycr048wΔ::KanMX4 ynr019wΔ::KanMX4 yor245cΔ::KanMX4 ynr008wΔ::KanMX4) expressing either murine DGAT1 or DGAT2 were incubated with diolein substrate in the absence or presence of 5 µM or different concentrations of TPE-5. Diolein substrate containing 0.2 mM diolein (*rac*-1,2/*sn*-1,3, 1/1) and 0.8 mM dioleoyl-phosphatidylcholine in Tris-buffer (50 mM, 20 mM MgCl_2_, pH 7.4), was prepared by sonication (Virsonic 475). Then, oleoyl-CoA (30 µM final concentration) and ^14^C-labeled oleoyl-CoA (55 µCi/µmol; 20 µM final concentration) were added. Substrate was added to samples and incubated for 10 min at 37°C. Lipids were extracted with chloroform/methanol (2:1, v/v) and separated on TLC using hexane/diethylether/acetic acid (70/29/1, v/v/v) as solvent. Bands corresponding to TAG were visualized by exposure to iodine vapor, scraped off, and radioactivity was determined by liquid scintillation counting (Tri-Carb 2300 TR).

Beta-oxidation rate quantifications:

Fatty acid β-oxidation was performed using metabolic radioactive labeling (Wang et al., 2009). AML12 cells were seeded in a 24 multiwell dish and were exposed to DMEM supplemented with 0.24 mmol/l fatty acid-free albumin (BSA), 0.5 mmol/l L-carnitine, 20 mmol/l HEPES, 0.5 oleic acid μCi/ml, 0.017 mmol/l) with 5 mmol/l glucose and 200 μM unlabeled OA, OA was used for labeling to avoid lipotoxicity (Muoio et al., 1999, Wang et al., 2012). After an overnight incubation, cells were washed twice with 1% bovine serum albumin in PBS and then incubated for an additional 6 h in MEM media with 1 mM carnitine without added OA (chase). After CO2 trapping, the incubation media were transferred to new tubes and assayed for labeled β-oxidation products (acid-soluble metabolites [ASMs]) (Muoio et al., 1999, Wang et al., 2012). The protein content of each sample was determined as described previously and used for normalization (Wang et al., 2012).

RNA-Seq library preparation and data analysis:

Experimental design

The design of the experiment included two major factors: activity and chemotype structure. We analyzed 3 pairs of active/inactive compounds. Each pair of active/inactive molecules represent one of 3 selected chemotypes or scaffolds: so it was active_#1/inactive_#1, active_#2/inactive_#2, active_#3/inactive_#3 based on phenotype activity. In addition 4 different controls without compounds were analyzed: 1) sample treated with vehicle (DMSO) with OA, which was used as a primary control for the treated compound samples, 2) sample treated with vehicle (DMSO) without OA, 3) non-treated sample with OA, 4) non-treated sample without OA. For gene expression all treated samples were prepared in triplicates, and non-treated in six replicates. In total we analyzed 3x2x3 (treated with compounds) + 2x3 (treated with vehicle) + 2x6 (controls) = 36 samples (GEO_Project GSE67503 and its description GEO_biosample_summary.xlx).

Cell culture

Drosophila S3 cells were grown in the presence of serum reduced medium (5% FCS) in Schneider’s Medium (PAN Biotech) in the presence or absence of the indicated small molecules at 1 µM concentration with or without 200 µM OA in 6-well plates. After 4 hours treatment, the cells were washed with PBS and used for RNA extraction (see below).

PolyA RNA isolation

We isolated total RNA using Qiagen RNAeasy Spin Mini Prep Kits (Qiagen, Hilden, Germany) according to the manufacturer’s description. We determined and recorded yield by UV spectroscopy using a NanoDrop device (Thermo). We used Dynabeads Oligo (dT)25 (Life Technologies, Carlsbad, CA, USA) to purify mRNA according to manufacturer’s instructions, except that we used 200 ng of total RNA adjusted the volume to 50 µl with dH2O, added 10 µl of the washed Dynabeads in a 50 µl binding buffer slurry, heated the samples at 65°C for 5min and immediately chilled on ice.

Molecular biology

To prepare RNA-Seq stranded libraries we modified an existing protocol (Wang et al., 2011). All steps were done using 96-well PCR plates (USA Scientific, Ocala, FL, USA). We incubated all reactions in a Tetrad PTC-225 Thermal Cycler (MJ Research, Waltham, MA, USA). To clean up enzymatic reactions and size select library fragments at any of several steps below, we used 0.1% w/v carboxyl-modified Sera-Mag Magnetic Speed-beads (MagNA beads, Thermo Fisher Scientific, Waltham, MA, USA) in XP buffer [20% PEG 8000, 2.5 M NaCl, (Sigma Aldrich, St. Louis, MO, USA)] following the protocol described in (Rohland and Reich, 2012), except that we incubated samples with beads for 10 min and used 80% ethanol for washing. For cleaning without size-selection we used 1.6X MagNA beads in XP buffer (except where noted) and for selecting fragments > 200bp - 1X MagNA beads in XP buffer. Unless noted, we left the beads in the sample after each step. We used Alpaqua 96R Ring Magnet Plates (Alpaqua, Beverly, MA, USA) for magnetic bead separation. We measured RNA quantity with Quant-iTª RiboGreen and DNA with Quant-iTª PicoGreen (Invitrogen, Carlsbad, CA, USA) in 384-well plates, PS, Fluotrac 600, black (Greiner Bio-One Inc, Longwood, FL, USA) using a Gemini EM Fluorescence Microplate Reader (Molecular Devices, Sunnyvale, CA, USA) according to the manufacturer’s instructions. We used Agilent Bioanalyzer RNA chips and High Sensitivity DNA chips on the 2100 Bioanalyzer system (Agilent, Santa Clara, CA, USA) according to the manufacturer’s instructions and visually inspected electrophorograms for sharp peaks resulting in simple pass/fail calls based on signs of obvious degradation and to check for library size and adapter dimer contamination.

cDNA library preparation

We fragmented mRNA bound to Dynabeads at 94°C for 8 min in 16 µl of 1.25X first strand MMuLV RT buffer (New England Biolabs, Beverly, MA, USA), with 100 ng random primers (Invitrogen, Carlsbad, CA, USA), and 10 pg ERCC spike-in controls (Jiang et al., 2011) from pools 78A and 78B (Zook et al., 2012) obtained from Marc Salit (NIST, National Institute of Standards and Technology, Gaithersburg, MD, USA). We chilled the samples immediately on ice for 2 min and eluted from the beads. We transferred 15 µl of the eluate to fresh 96-well PCR plate. We added 5 µl of the first strand synthesis mixture [0.3 mM dNTPs, 5 mM DTT, and 10 U M-MuLV RT (New England Biolabs, Ipswich, MA, USA), and 0.5 U SuperRase-In (Life Technologies, Carlsbad, CA, USA)] to the fragmented RNA in the fragmentation buffer and performed a reverse transcription reaction. We bound RNA/cDNA hybrid with 32 µl MagNA bead XP buffer slurry, washed, and eluted samples in 16 µl of dH2O. We then performed a second strand synthesis with dUTP by adding 5 µl of 1X NEB buffer2 (New England Biolabs, Ipswich, MA, USA), with 1 mM each of dATP, dCTP, dGTP and 2 mM dUTP (Thermo Fisher Scientific, Waltham, MD, USA), 10 U DNA PolI, 2.5 U RNAseH, and 2.5 mM DTT (New England Biolabs, Ipswich, MA, USA) and incubated at 16°C for 5 hours. We rebound, washed, and eluted as above. We repaired ends by adding 4 µl of NEBNext End Repair Module (New England Biolabs, Ipswich, MA, USA) to the eluate following the manufacturer’s instructions. We then rebound samples to MagNA beads, washed, and eluted as above. Next we performed dA-tailing by adding 4 µl of 1X Blue Buffer, with 1 U Klenow 3’-5’ exo- (Enzymatics, Beverly, MA, USA) and 1 mM dATP, to the eluate and incubated at 37°C for 30min. We then rebound samples to MagNA beads and washed as above, eluted with 21 µl dH2O, and transferred 10 µl of cDNA without beads to each of two fresh plates. One plate was used in the following and one plate was frozen as a back-up. We ligated RNA Adapter Indexes AR001-AR016, AR018-AR023, AR025, and AR027 (Illumina, San Diego, CA, USA) to dsDNA by adding 1 µl of an adapter to each 10 µl sample and 13 µl of 1X Rapid Ligation Buffer with 30 U T4 DNA Ligase (Enzymatics, Beverly, MA, USA), and incubated at 25°C for 10 min. We stopped reactions with a final concentration of 0.01 M EDTA. We added 25 µl MagNA beads XP buffer slurry (final PEG = 13.6%), bound cDNA to the beads, washed and eluted in 30 µl of dH2O as above. We size-selected libraries by adding 1X XP buffer (30 µl), washed, eluted samples with 24 µl dH2O, and transferred 23 µl of cDNA without beads to fresh plate. 11.5 µl of the eluate was transferred to another plate and used in the next step; the remaining samples were frozen as a back-up. We mixed dsDNA product with 2.5 U (0.5 µl) of Uracil DNA Glycosylase (New England Biolabs, Ipswich, MA, USA) and 3 µl of PCR Primer Cocktail (Illumina, San Diego, CA, USA) and incubated at 37°C for 30 min to digest the second strand DNA. We added 15 µl 2X KAPA HiFi HotStart ReadyMix (Kapa Biosystems, Woburn, MA, USA) directly to the UDG digested DNA mixture and performed a PCR amplification with the following programmed cycle: 95°C for 2 min, followed by 12 cycles of 98°C for 20 sec, 65°C for 30 sec, and 72°C for 30 sec; then 72°C for 5 min. We purified product by adding 30 µl MagNa beads XP buffer slurry, eluted in 30 µl dH2O and transferred 29 µl of cDNA library without beads to a fresh plate as described above. To assay plate-level failure, we took 11 samples from each 96-well plate and examined electropherograms for the strong signal in the 300-350 bp library target size and weak signal in the 100-150 bp range (primer dimer products).

Sequencing, mapping, expression values calculation

We pooled 15 ng of each library with unique 18 indexes into 4 pools for multiplex sequencing. All multiplexed libraries were again quantified and checked for quality as above. We performed single-end 76 bp sequencing on a HiSeq2000 Sequencing System (Illumina, San Diego, CA, USA) according to the manufacturer. We mapped reads that passed Chastity (score > 0.6) base-calling filtering (Illumina CASAVA pipeline 1.8.2). Reads were mapped to the *Drosophila melanogaster* reference genome including ERCC spike-in controls sequences using Tophat 2 (v2.0.10) with parameters -g 1 --library-type fr-firststrand -G Dmel.FB5.57.ERCC.gtf (Trapnell et al., 2012). Genome reference (Dmel.FB5.57.ERCC.fa) and gene model (Dmel.FB5.57.ERCC.gtf) used for the analysis correspond to BDGP Release5 and FlyBase Release Dmel.FB5.57. We deleted chrU, chrUextra, from original .fasta and .gff files and added ERCC sequences and ERCC annotations. We used a Perl script to convert the .gff to a .gtf file and to make it compatible with Tophat 2 and other downstream software packages. To include all annotated genes and RNA species in the downstream analysis and HTSeq read counts we changed the feature type (third column in gff/gtf file formats) for miRNA genes from “miRNA” to “exon”, because it was the only one RNA species without exon annotation. We used bowtie (v.2-2.1.0) implemented in Tophat 2 to make Bowtie indexes for genome. We used samtools (v0.1.19) (Li et al., 2009) to make indexes for chromosomes and to sort, merge alignments, and for reads statistics. Mapped reads at the gene-level were counted by HT-Seq (0.5.4p1) (Anders et al., 2014) with parameter --stranded=reverse -i gene_id -t exon and the same annotation used in mapping.

All raw output .fastq files after Illumina sequencing and preprocessing together with library details are available from Gene Expression Omnibus (NCBI, GEO, http://www.ncbi.nlm.nih.gov/geo/) Databases, GEO_Project GSE67503; description GEO_biosample_summary.xlx.

We calculated and normalized gene expression levels for each replicate using Cufflinks (2.1.1) (Roberts et al., 2011) with output FPKM value for each gene. Statistics to determine differentially expressed genes between samples was done using Cuffdiff (2.1.1.) (Trapnell et al., 2013). FPKM values for each gene in each replicate and sample are presented in Tables S2 and S3. The CuffDiff output file with p and q values for each gene expression between treated with active/inactive compound and primary control (treated with vehicle) is available in GEO Project (GEO_Project # GSE67503 and Table S5).

Post-sequencing quality control

Index contamination check

We performed multiplex sequencing and one of the important technical issue which must be eliminated before downstream analysis is index contamination. Even after demultiplexing with Illumina software it is possible to check index contamination for each library using adapter dimer information.

We used the demultiplexed raw reads and assumed that if there was contamination at the time when adapters were added or inside adapter solution itself adapter dimers would be formed with heterogenous indexes besides homogenous ones, and thus unexpected index would be found along with expected index. We searched for all indexes used for libraries preparation in the raw reads and calculated the percentage of each index. We first checked if index with the highest percentage matched the expected one. Second, we determined a cutoff for index contamination because unexpected indexes can be normally detected in the library reads due to sequencing errors. Previously using 926 different RNA-Seq libraries obtained from single flies we calculated the percentage of each unexpected index in each library used in the pool and found the maximum percentage is 0.0038 (Oliver et al., unpublished data). Since there are 17 unexpected adapters for each library, we took 0.9354 ratio (1-0.0038 * 17=0.9354) as a cutoff for percentage of expected index versus unexpected ones, all 36 samples in this project have the ratio > 0.98 (GEO project # GSE67503).

Low-expression cutoff

Gene expression variability can be traced back to biological and technical causes. With decreasing expression levels variability due to technical reason becomes really significant and prevents biological conclusions. Moreover high variance of low-expressed genes may decrease overall correlation and lead to false positives (Subramaniam and Hsiao, 2012). It is also known that non-specific PolII activity and the existence of weak termination signals allows the transcription of inactive as well as non-coding regions resulting in low expression signals (van Bakel et al., 2010). It was recently published that these regions may have a functional role (Hangauer et al., 2013), but low expression will not allow us to analyze them in RNA-Seq analysis. To distinguish stable expressed genes and low–expressed ones or technical noise we made a cutoff based on detected expression of intergenic spacers which are supposed to be not expressed in general. We consider as intergenic spacer any chromosome region between boundaries of annotated gene models. Short regions (less than read length, 76 bp) were filtered. We performed mapping to these regions and FPKM calculation in the same way as for genes and based on FPKM distribution applied 95 percentile of intergenic regions expression FPKM=1.04 as a cutoff for true expression (Figure S3).

Repeatability check

To check the repeatability of our biological replicates we performed correlation analysis of gene expression after applying low-expression cut-off using Spearman methods. We removed replicates with correlation coefficient (rho) < 0.93 within all other replicates of the sample and those that have low correlation with replicates from other samples and represent outliers (Figure S3**)**. After filtering we calculated also correlation between samples (Figure S3). It was high indicating that no one sample is much different than others and we can conclude an absence of any obvious toxic effect of the treatment. We observed also that control samples not treated with any compounds correlated better with each other.

Data analysis

To analyze the data we used packages implemented in R and also online resources. Genes.fpkm_tracking (Cufflinks output) files were analyzed in principal component analysis (PCA) using R package CummeRbund (version 2.0.0) (Trapnell et al., 2012). For constructing heatmap we used FPKM expression values of only differentially expressed (DE) genes between samples treated with active compounds and control sample treated only with vehicle (DMSO) all samples were feeding with oleic acid. We added also DE genes between sample used as a primary control (vehicle_OA) and sample treated with vehicle but in the absence of oleic acid to determine difference in gene expression as a response to oleic acid. To make clustering of the genes on the heatmap we first scaled FPKM values to get row Z scores and then used k-means clustering method to determine the effective number of clusters. Number of clusters - 5 was chosen as an optimum using “elbow” and “silhouette” methods in R packages “cluster” and “HSAUR”. We used “gplots” package in R for heatmap construction with option scale=“column” to improve visualization.

The matrix with row Z scores was used for Modulated Modularity Clustering (MMC) (<http://mmc.gnets.ncsu.edu/>) (Stone and Ayroles, 2009). MMC analysis was done according to the software manual. To understand the function of DE genes and the clustering mode we converted FlyBase gene_id to ncbi gene_id and mapped to the *Drosophila melanogaster* specific KEGG pathways (<http://www.genome.jp/kegg/pathway.html>) (Kanehisa et al., 2014). We used a color coding (e.g. NCBI_gene_ID 41625, #pathway color, #background color) which corresponds to the different CTs or which marks pathways showing a common response for all three CTs. The analysis was performed according to the advanced pathway mapping tool description (KEGG Mapper – Search&Color Pathway). We used the REVIGO (<http://revigo.irb.hr/>) (Supek et al., 2011) and GOrilla (<http://cbl-gorilla.cs.technion.ac.il/>) (Eden et al., 2009) software with standard settings for geneontology-based enrichment analyses.

Tests for statistical significance:

For pairwise comparisons a two-sided homoscedastic T-Test was performed to test for statistical significance of quantitative differences. Multiple comparisons were determined by one-way ANOVA and Bonferroni’s *post-hoc* testing. The following significance levels are used: not significant (n.s.) p≥0.05; * p<0.05; ** p<0.01; *** p<0.001.

**Supplemental References**

Anders, S., Pyl, P.T., and Huber, W. (2014). HTSeq-a Python framework to work with high-throughput sequencing data. Bioinformatics (Oxford, England) *31*, 166-9.

Van Bakel, H., Nislow, C., Blencowe, B.J., and Hughes, T.R. (2010). Most “dark matter” transcripts are associated with known genes. PLoS Biology *8*, e1000371.

Buszczak, M., Lu, X., Segraves, W.A., Chang, T.Y., and Cooley, L. (2002). Mutations in the midway gene disrupt a Drosophila acyl coenzyme A: diacylglycerol acyltransferase. Genetics *160*, 1511–1518.

Cao., J., Zhou, Y., Peng, H., Huang, X., Stahler, S., Suri, V., Qadri, A., Gareski, T., Jones, J., Hahm, S., et al. (2011). Targeting the Acyl-CoA:diacylglycerol acyltransferase 1 (DGAT1) with small molecule inhibitors for the treatment of metabolic diseases. The Journal of Biological Chemistry *286(48)*, 41838-51.

Dow, R.L., Li, J.-C.C., Pence, M.P., Gibbs, E.M., LaPerle, J.L., Litchfield, J., Piotrowski, D.W., Munchhof, M.J., Manion, T.B., Zavadoski, W.J., et al. (2011). Discovery of PF-04620110, a Potent, Selective, and Orally Bioavailable Inhibitor of DGAT-1. ACS Medicinal Chemistry Letters *2*, 407–412.

Eden, E., Navon, R., Steinfeld, I., Lipson, D., and Yakhini, Z. (2009). GOrilla: a tool for discovery and visualization of enriched GO terms in ranked gene lists. BMC Bioinformatics *10*, 48.

Fox, B.M., Sugimoto, K., Iio, K., Yoshida, A., Zhang, J.K., Li, K., Hao, X., Labelle, M., Smith, M.-L.L., Rubenstein, S.M., et al. (2014). Discovery of 6-phenylpyrimido[4,5-b][1,4]oxazines as potent and selective acyl CoA:diacylglycerol acyltransferase 1 (DGAT1) inhibitors with in vivo efficacy in rodents. J. Med. Chem. *57*, 3464–3483.

Grönke, S., Beller, M., Fellert, S., Ramakrishnan, H., Jäckle, H., and Kühnlein, R.P. (2003). Control of fat storage by a Drosophila PAT domain protein. Current Biology : CB *13*, 603–606.

Grönke, S., Mildner, A., Fellert, S., Tennagels, N., Petry, S., Müller, G., Jäckle, H., and Kühnlein, R.P. (2005). Brummer lipase is an evolutionary conserved fat storage regulator in Drosophila. Cell Metab. *1*, 323–330.

Hangauer, M.J., Vaughn, I.W., and McManus, M.T. (2013). Pervasive transcription of the human genome produces thousands of previously unidentified long intergenic noncoding RNAs. PLoS Genetics *9*, e1003569.

Hauner, H., Skurk, T., and Wabitsch, M. (2001). Cultures of human adipose precursor cells. Methods in Molecular Biology (Clifton, N.J.) *155*, 239–247.

Henry, R.R., Abrams, L., Nikoulina, S., and Ciaraldi, T.P. (1995). Insulin action and glucose metabolism in nondiabetic control and NIDDM subjects. Comparison using human skeletal muscle cell cultures. Diabetes *44*, 936–946.

Hildebrandt, A., Bickmeyer, I., and Kühnlein, R. (2011). Reliable Drosophila Body Fat Quantification by a Coupled Colorimetric Assay. PLoS ONE *6*, e23796.

Igal, R.A., Wang, P., and Coleman, R.A. (1997). Triacsin C blocks de novo synthesis of glycerolipids and cholesterol esters but not recycling of fatty acid into phospholipid: evidence for functionally separate pools of acyl-CoA. Biochem. J. *324 ( Pt 2)*, 529–534.

Inglese, J., Auld, D.S., Jadhav, A., Johnson, R.L., Simeonov, A., Yasgar, A., Zheng, W., and Austin, C.P. (2006). Quantitative high-throughput screening: a titration-based approach that efficiently identifies biological activities in large chemical libraries. Proceedings of the National Academy of Sciences of the United States of America *103*, 11473–11478.

Jiang, L., Schlesinger, F., Davis, C.A., Zhang, Y., Li, R., Salit, M., Gingeras, T.R., and Oliver, B. (2011). Synthetic spike-in standards for RNA-seq experiments. Genome Research *21*, 1543–1551.

Kanehisa, M., Goto, S., Sato, Y., Kawashima, M., Furumichi, M., and Tanabe, M. (2014). Data, information, knowledge and principle: back to metabolism in KEGG. Nucleic Acids Research *42*, D199–205.

Li, H., Handsaker, B., Wysoker, A., Fennell, T., Ruan, J., Homer, N., Marth, G., Abecasis, G., and Durbin, R. (2009). The Sequence Alignment/Map format and SAMtools. Bioinformatics (Oxford, England) *25*, 2078–2079.

Liu, J., Gorski, J.N., Gold, S.J., Chen, D., Chen, S., Forrest, G., Itoh, Y., Marsh, D.J., McLaren, D.G., Shen, Z., et al. (2013). Pharmacological inhibition of diacylglycerol acyltransferase 1 reduces body weight and modulates gut peptide release--potential insight into mechanism of action. Obesity (Silver Spring, Md.) *21*, 1406–1415.

McCoull, W., Addie, M.S., Birch, A.M., Birtles, S., Buckett, L.K., Butlin, R.J., Bowker, S.S., Boyd, S., Chapman, S., Davies, R.D., et al. (2012). Identification, optimisation and in vivo evaluation of oxadiazole DGAT-1 inhibitors for the treatment of obesity and diabetes. Bioorganic & Medicinal Chemistry Letters *22*, 3873–3878.

Muoio, D.M., Seefeld, K., Witters, L.A., and Coleman, R.A. (1999). AMP-activated kinase reciprocally regulates triacylglycerol synthesis and fatty acid oxidation in liver and muscle: evidence that sn-glycerol-3-phosphate acyltransferase is a novel target. The Biochemical Journal *338 ( Pt 3)*, 783–791.

Ritz, C., and Streibig, J. (2005). Bioassay Analysis using R. Journal of Statistical Software *12*.

Roberts, A., Trapnell, C., Donaghey, J., Rinn, J.L., and Pachter, L. (2011). Improving RNA-Seq expression estimates by correcting for fragment bias. Genome Biology *12*, R22.

Rohland, N., and Reich, D. (2012). Cost-effective, high-throughput DNA sequencing libraries for multiplexed target capture. Genome Research *22*, 939–946.

Serrano-Wu, M.H., Kwak, Y., Coppola, G., Foster, C., Gilmore, T., Gong, Y., He, G., Hou, Y., Kantor, A., Li, J., et al. (2012). Discovery of a DGAT1 inhibitor with robust suppression of postprandial triglyceride levels in humans. (San Diego, CA, USA).

Shirazi, F., Farmakiotis, D., Yan, Y., Albert, N., Kim-Anh, D., and Kontoyiannis, D.P. (2014). Diet modification and metformin have a beneficial effect in a fly model of obesity and mucormycosis. PloS One *9*, e108635.

Stone, E.A., and Ayroles, J.F. (2009). Modulated modularity clustering as an exploratory tool for functional genomic inference. PLoS Genetics *5*, e1000479.

Subramaniam, S., and Hsiao, G. (2012). Gene-expression measurement: variance-modeling considerations for robust data analysis. Nature Immunology *13*, 199–203.

Supek, F., Bošnjak, M., Škunca, N., and Šmuc, T. (2011). REVIGO summarizes and visualizes long lists of gene ontology terms. PloS One *6*, e21800.

Trapnell, C., Roberts, A., Goff, L., Pertea, G., Kim, D., Kelley, D.R., Pimentel, H., Salzberg, S.L., Rinn, J.L., and Pachter, L. (2012). Differential gene and transcript expression analysis of RNA-seq experiments with TopHat and Cufflinks. Nature Protocols *7*, 562–578.

Trapnell, C., Hendrickson, D.G., Sauvageau, M., Goff, L., Rinn, J.L., and Pachter, L. (2013). Differential analysis of gene regulation at transcript resolution with RNA-seq. Nature Biotechnology *31*, 46–53.

Wang, H., Hu, L., Dalen, K., Dorward, H., Marcinkiewicz, A., Russell, D., Gong, D., Londos, C., Yamaguchi, T., Holm, C., et al. (2009). Activation of Hormone-sensitive Lipase Requires Two Steps, Protein Phosphorylation and Binding to the PAT-1 Domain of Lipid Droplet Coat Proteins. Journal of Biological Chemistry *284*, 32116–32125.

Wang, H., Sreenivasan, U., Sreenevasan, U., Hu, H., Saladino, A., Polster, B.M., Lund, L.M., Gong, D.W., Stanley, W.C., and Sztalryd, C. (2012). Perilipin 5, a lipid droplet-associated protein, provides physical and metabolic linkage to mitochondria. Journal of Lipid Research *52*, 2159–2168.

Wang, Y., Ghaffari, N., Johnson, C.D., Braga-Neto, U.M., Wang, H., Chen, R., and Zhou, H. (2011). Evaluation of the coverage and depth of transcriptome by RNA-Seq in chickens. BMC Bioinformatics *12 Suppl 10*, S5.

Yamamoto, T., Yamaguchi, H., Miki, H., Kitamura, S., Nakada, Y., Aicher, T.D., Pratt, S.A., and Kato, K. (2011). A novel coenzyme A:diacylglycerol acyltransferase 1 inhibitor stimulates lipid metabolism in muscle and lowers weight in animal models of obesity. European Journal of Pharmacology *650*, 663–672.

Yeh, V.S., Beno, D.W., Brodjian, S., Brune, M.E., Cullen, S.C., Dayton, B.D., Dhaon, M.K., Falls, H.D., Gao, J., Grihalde, N., et al. (2012). Identification and preliminary characterization of a potent, safe, and orally efficacious inhibitor of acyl-CoA:diacylglycerol acyltransferase 1. Journal of Medicinal Chemistry *55*, 1751–1757.

Zhao, G., Souers, A.J., Voorbach, M., Falls, H.D., Droz, B., Brodjian, S., Lau, Y.Y., Iyengar, R.R., Gao, J., Judd, A.S., et al. (2008). Validation of diacyl glycerolacyltransferase I as a novel target for the treatment of obesity and dyslipidemia using a potent and selective small molecule inhibitor. Journal of Medicinal Chemistry *51*, 380–383.

Zook, J.M., Samarov, D., McDaniel, J., Sen, S.K., and Salit, M. (2012). Synthetic spike-in standards improve run-specific systematic error analysis for DNA and RNA sequencing. PloS One *7*, e41356.
